# Botrytis cinerea Transcription Factor BcXyr1 Regulates (Hemi-)Cellulase Production and Fungal Virulence

**DOI:** 10.1128/msystems.01042-22

**Published:** 2022-12-05

**Authors:** Liang Ma, Tong Liu, Ke Zhang, Haojie Shi, Lei Zhang, Gen Zou, Amir Sharon

**Affiliations:** a The Key Laboratory for Quality Improvement of Agricultural Products of Zhejiang Province, College of Advanced Agricultural Sciences, Zhejiang A&F University, Hangzhou, People’s Republic of China; b State Key Laboratory for Conservation and Utilization of Bio-Resource in Yunnan, Yunnan University, Kunming, Yunnan, People’s Republic of China; c Yunnan Tobacco Quality Inspection & Supervision Station, Kunming, People’s Republic of China; d DeepBiome. Co. Ltd., Shanghai, People’s Republic of China; e Shanghai Key Laboratory of Agricultural Genetics and Breeding, Institute of Edible Fungi, Shanghai Academy of Agricultural Sciences, Shanghai, People’s Republic of China; f School of Plant Sciences and Food Security, Tel Aviv University, Tel Aviv, Israel; University of Dundee

**Keywords:** *Botrytis cinerea*, transcription factor, BcXyr1, (hemi-)cellulase, virulence, PCWDEs, BcExl1

## Abstract

Botrytis cinerea is an agriculturally notorious plant-pathogenic fungus with a broad host range. During plant colonization, *B. cinerea* secretes a wide range of plant-cell-wall-degrading enzymes (PCWDEs) that help in macerating the plant tissue, but their role in pathogenicity has been unclear. Here, we report on the identification of a transcription factor, BcXyr1, that regulates the production of (hemi-)cellulases and is necessary for fungal virulence. Deletion of the *bcxyr1* gene led to impaired spore germination and reduced fungal virulence and reactive oxygen species (ROS) production *in planta*. Secreted proteins collected from the *bcxyr1* deletion strain displayed a weaker cell-death-inducing effect than the wild-type secretome when infiltrated to Nicotiana benthamiana leaves. Transcriptome sequencing (RNA-seq) analysis revealed 41 genes with reduced expression in the Δ*bcxyr1* mutant compared with those in the wild-type strain, of which half encode secreted proteins that are particularly enriched in carbohydrate-active enzyme (CAZyme)-encoding genes. Among them, we identified a novel putative expansin-like protein that was necessary for fungal virulence, supporting the involvement of BcXyr1 in the regulation of extracellular virulence factors.

**IMPORTANCE** PCWDEs are considered important components of the virulence arsenal of necrotrophic plant pathogens. However, despite intensive research, the role of PCWDEs in the pathogenicity of necrotrophic phytopathogenic fungi remains ambiguous. Here, we demonstrate that the transcription factor BcXyr1 regulates the expression of a specific set of secreted PCWDE-encoding genes and that it is essential for fungal virulence. Furthermore, we identified a BcXyr1-regulated expansin-like gene that is required for fungal virulence. Our findings provide strong evidence for the importance of PCWDEs in the pathogenicity of *B. cinerea* and highlight specific PCWDEs that might be more important than others.

## INTRODUCTION

Botrytis cinerea is a well-known plant-pathogenic fungus that is capable of infecting more than 1,000 plant species and is responsible for huge economic losses worldwide (1). During the infection process, *B. cinerea* secrets a wide range and large amounts of plant-cell-wall-degrading enzymes (PCWDEs) that macerate the plant tissue. Due to their high abundance and role in tissue degradation, PCWDEs have been considered virulence factors and have been studied for close to a century (2–4). However, despite intensive studies, there is no direct evidence for a role in pathogenicity for the vast majority of the *B. cinerea* PCWDEs (5–7).

The plant cell wall, which is composed mainly of cellulose, hemicellulose, and pectin, is among the first structures that phytopathogenic fungi encounter when colonizing plant tissues (8). The plant cell wall is a potential source of nutrients for pathogens, but intact walls are also an important barrier for effective defense against phytopathogenic fungi (7). The secreted PCWDEs contain a mixture of enzymes capable of degrading the different types of plant cell wall sugar polymers. The carbohydrate-active enzymes (CAZymes) of classes carbohydrate esterases (CEs), glycoside hydrolases (GHs), and polysaccharide lyases (PLs) are often known as plant-cell-wall-degrading enzymes due to their important roles in plant biomass decomposition (9). Necrotrophic plant pathogens, which derive nutrients from dead tissue, are characterized by the highest number and diversity of CAZymes in their genomes (5, 9). In *B. cinerea*, there are 275 putatively secreted CAZymes, and it is intuitive to assume that at least some of them contribute to the ability of the fungus to infect a wide range of host plants (8). A large number of *B. cinerea* PCWDEs have been mutated and analyzed; however, a clear effect on fungal virulence was demonstrated only in a small number of cases. The lack of a clear phenotype was attributed to functional redundancy, which interferes with efforts to identify PCWDEs as virulence determinants (10). Previously reported PCWDEs that contribute to fungal virulence include Bcpg1, Bcpg2, Xyn11A, BcAra1, BcXyl1, BcCBH, and BcEG (11–16). However, a recent study showed inconsistent results that no significant virulence defects were observed in mutants lacking Xyn11A or Xyl1, and Bcpg1 and Bcpg2 are the exclusive PCWDEs that were verified to be involved in the fungal virulence (17). Therefore, the general role of *Botrytis* PCWDEs in pathogenicity remains unclear.

In fungi, expression of PCWDE-encoding genes is usually governed by specific transcription factors. In Trichoderma reesei, the Xyr1 transcription factor functions as the main regulator of PCWDE-encoding genes (18–20), and its homologues are assumed to have a similar function in different fungi (21). Compared with saprophytic fungi, phytopathogenic fungi received less attention concerning the regulatory landscape associated with PCWDE production. Studies of the Xyr1/XlnR homologues in plant-pathogenic fungi showed functional differences between species. For example, Xyr1 regulated both xylanase and cellulase production in Fusarium graminearum, while XlnR in Fusarium oxysporum affected only xylanase gene expression (22, 23). In contrast, in Magnaporthe oryzae, XlnR regulated the pentose catabolic pathway but not genes encoding (hemi-)cellulolytic enzymes (24).

In order to elucidate the function of the Xyr1/XlnR homologue in *B. cinerea* and in particular its role in pathogenicity, we identified and analyzed *bcxyr1*, a *B. cinerea* homologue of *T. reesei xyr1*. Deletion of *bcxyr1* resulted in reduced fungal virulence, and transcriptome sequencing (RNA-seq) analysis revealed that BcXyr1 positively regulates a total of 22 genes encoding predicted secreted proteins, which were enriched in CAZyme-encoding genes. Among them, a putative expansin-like protein was shown to be required for fungal virulence. These results unveil the previously unknown function of the PCWDE regulator BcXyr1 and its downstream target gene which contributes to virulence.

## RESULTS

### BcXyr1 is required for (hemi-)cellulase production.

**FIG 1:**
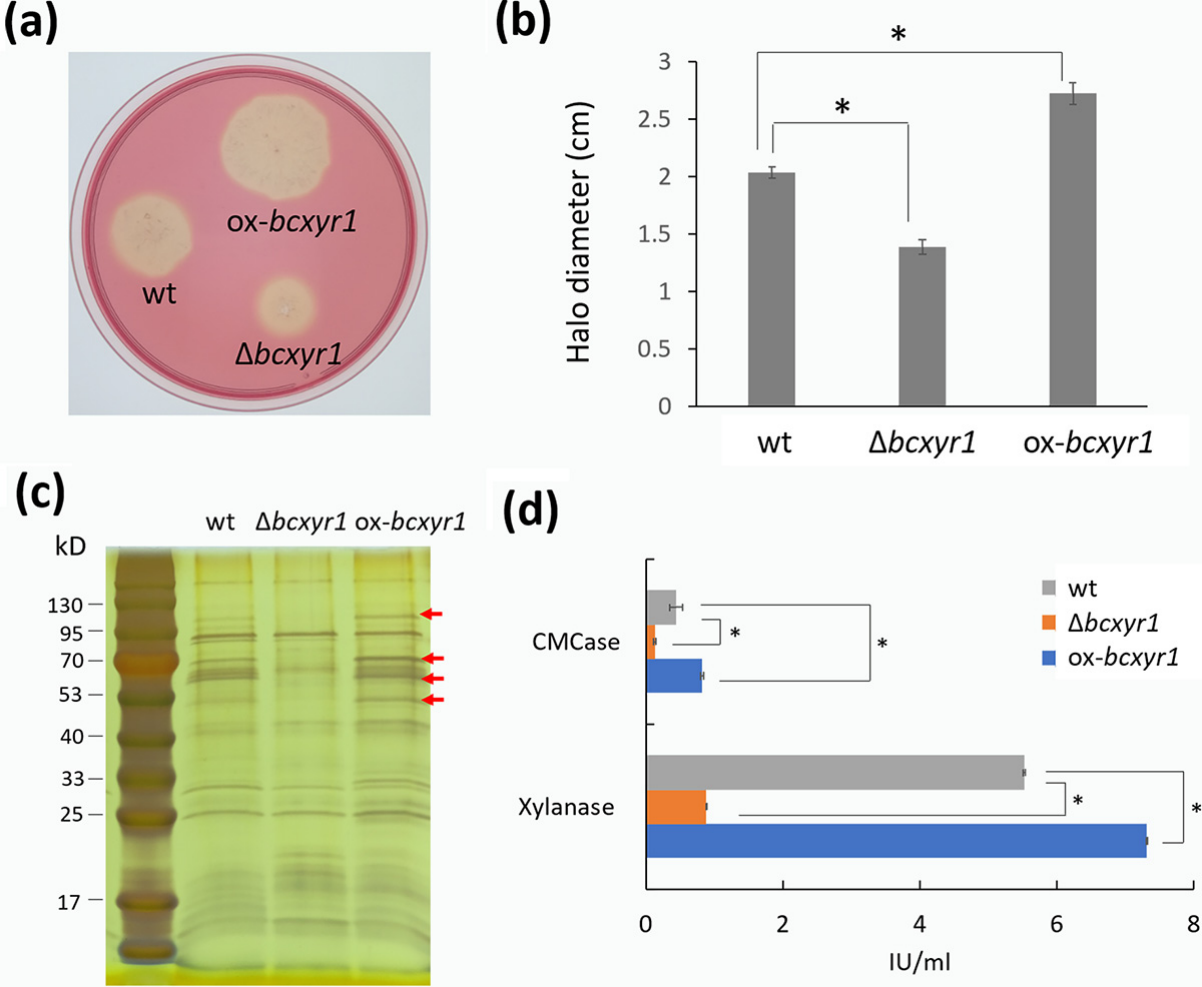
*bcxyr1* is required for (hemi)cellulase production in *B. cinerea*. (a) Colonies were initiated by placing a 5-μL droplet containing 500 spores of wild-type (wt), Δ*bcxyr1*, or ox-*bcxyr1* strains onto a CMC plate, and the plates were incubated for 4 days and then stained by Congo red. (b) Average halo diameter of each strain after incubation for 4 days. (c) Three strains were grown in liquid Gamborg medium supplemented with 2% rice straw powder as a (hemi)cellulase inducer, and after 1 day of growth, 20 μL of culture supernatant of each strain was subjected to SDS-PAGE and then silver staining. (d) Xylanase activity and CMCase activity of culture supernatant samples of c were measured. Values that are statistically significantly different (*P < *0.01) by two-tailed Student's *t* test from the wild-type values are indicated by an asterisk.

A BLAST search of the *B. cinerea* genome ranked BcXyr1 (Bcin12g02060) as the closest homologue of *T. reesei* Xyr1 (GenBank AAO33577.1). The *bcxyr1* gene encodes a predicted protein of 966 amino acids with a typical Zn_2_Cys_6_ fungal-type DNA-binding domain. To investigate the function of BcXyr1, we generated and characterized *bcxyr1* deletion (Δ*bcxyr1*) and overexpression (ox*-bcxyr1*) strains. Real-time PCR analysis showed a lack of transcript in the Δ*bcxyr1* strain and a 5.4-fold increase of *bcxyr1* expression in the ox-*bcxyr1* strain (see Fig. S1d in the supplemental material). Since the transcription factor Xyr1/XlnR is a major regulator in cellulose and xylan degradation in various fungi (21), we tested if BcXyr1 has a similar function. To this end, we performed the carboxymethyl cellulose (CMC)-containing Gamborg B5 plate assay and beechwood xylan-containing Gamborg B5 plate assay to compare the cellulase- and xylanase-producing ability among different strains. After incubation for 4 days, the Δ*bcxyr1* strain formed a significantly smaller halo diameter (1.39 cm) than the wild-type strain (2.04 cm), while the ox-*bcxyr1* strain formed a larger halo (2.73 cm), confirming that BcXyr1 functions as an activator of cellulase production (Fig. 1a and b). When their xylanase-producing abilities were compared, however, there was difficulty in measuring the halo zones since all the three strains (wild type, Δ*bcxyr1*, and ox-*bcxyr1*) formed irregularly shaped colonies when growing in the beechwood xylan-containing Gamborg B5 plates. For better assessment of (hemi-)cellulase production, fungi were grown in Gamborg liquid medium supplemented with 2% rice straw powder as a (hemi-)cellulase-inducing carbon source. The protein patterns of the culture supernatant displayed a distinguishable difference between the wild type and the mutant strains. After incubation for 24 h, the protein bands in the Δ*bcxyr1* sample were not only fewer but also lighter than those of the wild-type protein sample, while the sample of ox-*bcxyr1* showed an opposite trend with stronger bands (Fig. 1c). Moreover, compared with the wild type, the carboxymethyl cellulase (CMCase) activity and xylanase activity of Δ*bcxyr1* were decreased by 69% and 84%, respectively. In contrast, the ox-*bcxyr1* strain showed an 86% increase of CMCase and 32% increase of xylanase activity (Fig. 1d). These results confirmed that BcXyr1 regulates the expression of (hemi-)cellulase genes, similar to its homologues in some other fungi, such as Xyr1 in *T. reesei* (18, 19) and Xyr1 in Fusarium graminearum (22).

10.1128/msystems.01042-22.1 FIG S1 Generation of *B. cinerea* mutants. (a) The deletion cassette used to generate the Δ*bcxyr1* mutant contained the hygromycin resistance gene (*hph*) flanked by the upstream region and the downstream region of the *bcxyr1* gene. (b) The *bcxyr1* complementation cassette contains the upstream and the downstream regions of the *bcxyr1* locus, the *bcxyr1* ORF, the *Tcel5a* termination signal, and the nourseothricin resistance gene (*nr*). (c) The *bcxyr1* overexpression cassette contains the 3′ part of the *bcgapdh* ORF and its termination sequence, the *bcxyr1* ORF under a H2B promoter flanked by a *hph* gene, and a 3′ flank fragment adjacent to the *bcgapdh* termination sequence. (d) Expression levels of *bcxyr1* in mutants were compared to the wild-type strain, and *bcgpdh* was used as a control gene to normalize data. The primers used to verify the transformants are indicated (arrows). Values that are statistically significantly different (*P < *0.01) by two-tailed Student’s *t*-test from the wild-type values are indicated by an asterisk. Download FIG S1, TIF file, 0.5 MB. Copyright © 2022 Ma et al. 2022 Ma et al. https://creativecommons.org/licenses/by/4.0/ This content is distributed under the terms of the Creative Commons Attribution 4.0 International license.

### *bcxyr1* is required for spore germination but dispensable for sporulation and mycelial growth.

**FIG 2:**
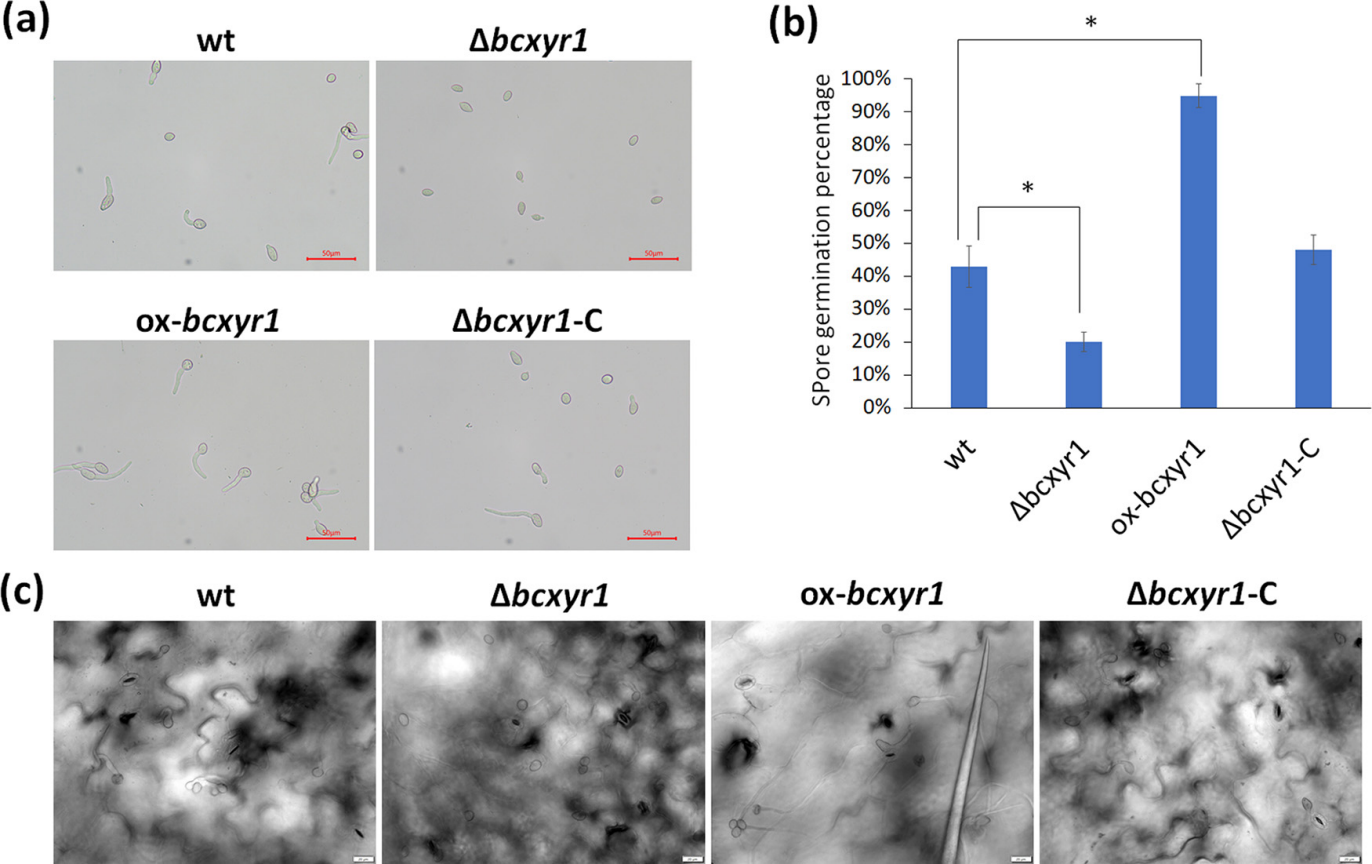
Deletion of *bcxyr1* affects spore germination. (a) Spore germination in CD + 1% sucrose medium. Spores were diluted to 10^5^/mL in liquid CD + 1% sucrose medium. A total of 15 μL of spore suspension of each strain was pipetted onto glass coverslips and incubated for 6 h. (b) Spore germination percentage of each sample after incubation for 6 h was counted under an inverted microscope. Four replicates were used for each sample. Values that are statistically significantly different (*P < *0.01) by two-tailed Student’s *t* test from the wild-type values are indicated by an asterisk. (c) A total of 7.5 μL of spore suspension (10^5^/mL) in liquid CD + 1% sucrose medium was inoculated onto *Arabidopsis* leaves, images were taken at 8 hpi, and the scale bar indicates 20 μm.

Deletion of *bcxyr1* had no effect on sporulation and mycelial growth (see Fig. S2 in the supplemental material). However, the *bcxyr1* deletion strain had 50% reduced germination rates compared with the wild-type strain after incubation for 6 h on glass coverslips, whereas the overexpression strain had an opposite effect (Fig. 2a and b). Similarly, more than 70% of the wild-type spores germinated on *Arabidopsis* leaves at 8 h postinoculation (hpi), in comparison to almost no germination in the Δ*bcxyr1* strain and close to 100% spore germination in the ox-*bcxyr1* strain (Fig. 2c).

10.1128/msystems.01042-22.2 FIG S2 *bcxyr1* is not required for sporulation and mycelial radial growth in *B. cinerea*. (a) Spore suspensions (10^5^/mL, 5 μL) of *B. cinerea* wild-type strain 05.10 and the Δ*bcxyr1* strain were inoculated onto PDA plates, and the spore number of each plate was determined at 7 dpi. (b) Spore suspensions (10^5^/mL, 5 μL) of *B. cinerea* wild-type strain and the Δ*bcxyr1* strain were inoculated onto PDA plates and CD + 2% sucrose plates. Colony diameters were determined at 3 dpi. Columns marked by different letters represented statistical differences (*P < *0.01). Download FIG S2, TIF file, 0.8 MB. Copyright © 2022 Ma et al. 2022 Ma et al. https://creativecommons.org/licenses/by/4.0/ This content is distributed under the terms of the Creative Commons Attribution 4.0 International license.

### BcXyr1 is essential for virulence.

**FIG 3:**
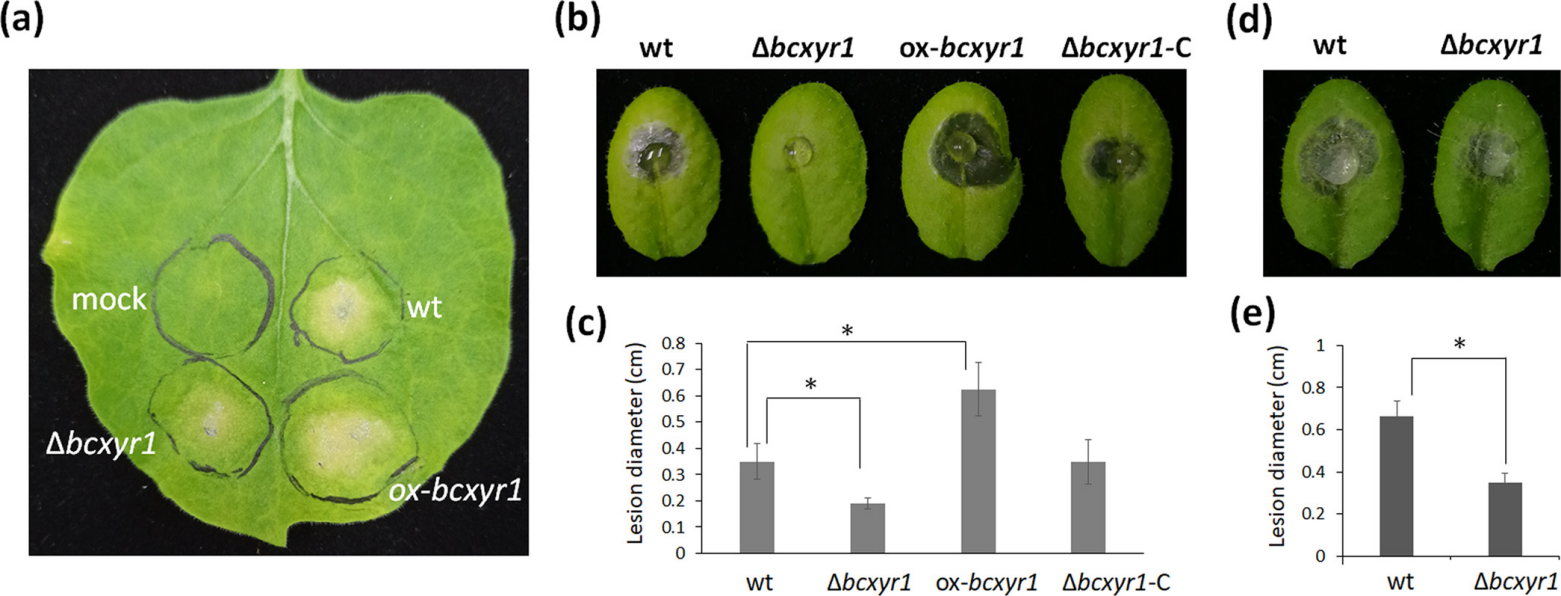
*bcxyr1* is required for fungal virulence. (a) The culture supernatants (20 μg/mL protein in PBS) from strains which grew 24 h in Gamborg medium supplemented with 2% rice straw powder were infiltrated into N. benthamiana leaves, and necrosis was observed 5 days after infiltration. PBS was used as a mock control. The black circles indicate the infiltrated area. (b) *Arabidopsis* leaves were inoculated with 7.5-μL droplets of spore suspension (10^5^ spores/mL in liquid CD + 1% sucrose medium), and typical lesions were developed at 80 hpi. (c) Average lesion size of sample b, and data are the means plus SD for eight lesions from three plants. (d) *Arabidopsis* leaves were inoculated with mycelial plugs (3 mm in diameter) from a CD + 1% sucrose agar plate, and typical lesions were developed at 34 hpi. (e) Average lesion size of sample d, and data are the means plus SD for eight lesions from three plants. Values that are statistically significantly different (*P < *0.01) by two-tailed Student’s *t* test from the wild-type values are indicated by an asterisk.

Certain *B. cinerea* xylan-degrading enzymes have a cell-death-inducing activity (13, 15). Since (hemi-)cellulase production is significantly impaired in the Δ*bcxyr1* strain, it is possible that deletion of *bcxyr1* also affects the secretion of cell-death-inducing proteins (CDIPs). To this end, the culture supernatants from strains which grew 24 h in Gamborg medium plus 2% rice straw powder (Fig. 1) were collected and filtered. The protein concentration in the filtrates was determined and diluted to 20 μg/mL in phosphate-buffered saline (PBS) and then injected into N. benthamiana leaves. After 5 days, treatment of N. benthamiana leaves with culture filtrate from the ox-*bcxyr1* strain led to the strongest cell death, while treatment with culture filtrate from the Δ*bcxyr1* strain resulted in the weakest phenotype (Fig. 3a). The difference of cell death-inducing effect between strains suggests that there are secreted cell-death-inducing factors, which are regulated by BcXyr1.

A pathogenicity assay on Arabidopsis thaliana leaves showed reduced and increased lesion sizes for the Δ*bcxyr1* strain and the ox-*bcxyr1* strain, respectively; at 80 hpi, the Δ*bcxyr1* mutant produced an average lesion of 0.19 cm compared with 0.35 cm by the wild-type and 0.63 cm by the ox-*bcxyr1* strains (Fig. 3b and c). Infection with mycelial plugs reproduced these phenotypes (Fig. 3d and e), which ruled out a possible effect of spore germination rates. We obtained similar results by infection of tomato and cucumber leaves (see Fig. S3 in the supplemental material), confirming that BcXyr1 is necessary for the full virulence of this fungus.

10.1128/msystems.01042-22.3 FIG S3 Cucumber leaves (a) and tomato leaves (b) were inoculated with 7.5-μL droplets of spore suspensions containing 10^5^ spores/mL in liquid CD + 1% sucrose medium, and typical lesions were developed at 4 dpi. Values that are statistically significantly different (*P < *0.01) by a two-tailed Student’s *t*-test from the wild-type values are indicated by an asterisk. Download FIG S3, TIF file, 1.5 MB. Copyright © 2022 Ma et al. 2022 Ma et al. https://creativecommons.org/licenses/by/4.0/ This content is distributed under the terms of the Creative Commons Attribution 4.0 International license.

### *bcxyr1* affects hyphal orientation and ROS induction in *Arabidopsis*.

**FIG 4:**
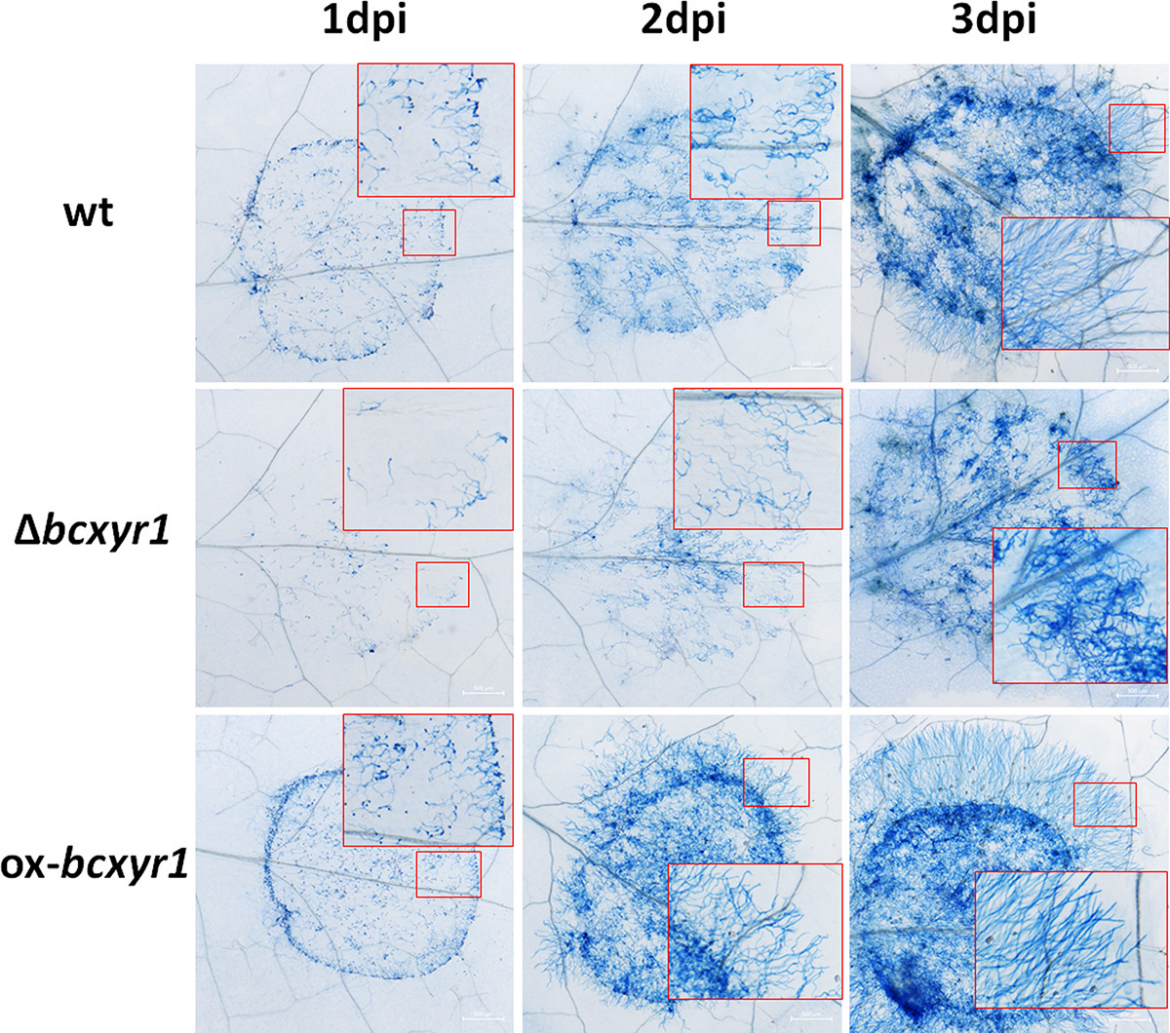
*Arabidopsis* leaves were inoculated with 7.5-μL droplets of spore suspension (10^5^ spores/mL in liquid CD + 1% sucrose medium). Leaves were cut at the designated time points, and samples were stained with lactophenol trypan blue. The large images show the entire lesion; squares show hyphal orientation and structure at the colony edge. Pictures were taken with the same magnification, and scale bar indicates 500 μm.

To gain insight into the infection process, we stained infected *Arabidopsis* leaf tissue with lactophenol trypan blue, which stains dead cells (25). Although mostly germlings were observed for all the three strains in the inoculation spot at 1 day postinoculation (dpi) (Fig. 4), visually less fungal biomass was observed in leaves that were inoculated with the Δ*bcxyr1* strain. This difference is probably owing to the retarded spore germination of the Δ*bcxyr1* strain on *Arabidopsis* leaves since ungerminated spores can be easily washed off the plant surface during staining while germinated spores attach strongly through secretion of an extracellular matrix during germination (26). The ox-*bcxyr1* strain developed oriented hyphae toward the periphery of the lesion at 2 dpi, and although the wild type formed oriented hyphae at 3 dpi, the ox-*bcxyr1* showed a clearly far more ordered and aggressive hyphal pattern. Since the hyphal orientation is associated with the transition from local necrosis to a spreading lesion (27), a delay in hyphal orientation suggests weakened fungal virulence, which accords with the results of the pathogenicity test (Fig. 3b and c).

As a response to pathogen infestation, the plant releases large amounts of ROS to counteract the pathogen, known as oxidative burst (28, 29). It was shown that *B. cinerea* exploits this plant defense reaction and even contributes to oxidative burst by forming its own ROS (29). To check if H_2_O_2_ production correlates with BcXyr1-regulated fungal infection, 3,3'-diaminobenzidine (DAB) staining assay was performed to trace H_2_O_2_ production during the *B. cinerea*-*Arabidopsis* interaction. Plants that were inoculated with the ox-*bcxyr1* strain accumulated more H_2_O_2_ than the wild-type-inoculated leaves at 24 hpi, while there was hardly any H_2_O_2_ detected in the leaf inoculated with the Δ*bcxyr1* strain at the same time point. Similar results were observed at 48 hpi that showed the highest accumulation of H_2_O_2_ in the ox-*bcxyr1*-inoculated leaves and the lowest accumulation in the Δ*bcxyr1*-inoculated leaves (see Fig. S4 in the supplemental material). Therefore, the H_2_O_2_ level well reflected the BcXyr1-associated fungal virulence.

10.1128/msystems.01042-22.4 FIG S4 H_2_O_2_ production correlates with BcXyr1-regulated fungal infection. Spores were diluted to 10^5^/mL in liquid CD + 1% sucrose medium, and 7.5 μL of spore suspension was inoculated onto *Arabidopsi*s leaves. H_2_O_2_ production was detected by 3,3-diaminobenzidine (DAB) staining at 24 and 48 dpi. The brown intensity of DAB staining was quantified using ImageJ software. The scale bar indicates 1,000 μm. Values that are statistically significantly different (*P < *0.01) by two-tailed Student’s *t*-test from the wild-type values are indicated by an asterisk. Download FIG S4, TIF file, 5.4 MB. Copyright © 2022 Ma et al. 2022 Ma et al. https://creativecommons.org/licenses/by/4.0/ This content is distributed under the terms of the Creative Commons Attribution 4.0 International license.

### CAZymes are regulated by the BcXyr1.

In preparation for RNA-seq, *B. cinerea* wild-type strain B05.10 and Δ*bcxyr1* strains were precultured in liquid malt medium for 20 h, and then the mycelium was collected, washed, and transferred into liquid CD + 1% sucrose medium for another 7 h. Then samples were prepared for RNA isolation and further sequencing. After sequence quality control and mapping, the number of clean reads per sample ranged from 20.73 to 21.93 million, and resulted in clean bases per sample between 6.2 Gbp and 6.56 Gbp. We found 95 genes that were differentially expressed in the Δ*bcxyr1* strain compared with the wild-type strain, of which 41 were downregulated in Δ*bcxyr1* (see Table S1 in the supplemental material). These results were further validated by quantification of the expression of nine selected genes of the wild type and the Δ*bcxyr1* strain with real-time quantitative PCR (qRT-PCR) (see Table S2 in the supplemental material). We also analyzed gene expression in the ox-*bcxyr1* strain and found that the ox-*bcxyr1* strain and the Δ*bcxyr1* strain showed opposite expression trends in seven out of the nine selected genes. The two other genes (Bcin02g07640 and Bcin14g05500) were both regulated in the same trend in the deletion and the overexpression strains. A similar observation was reported in *T. reesei*, in which the *Xyr1* overexpression strain did not show an opposite expression trend in all the target genes compared with a Xyr1-deficient strain (30); however, the reason for this phenomenon is currently unclear.

10.1128/msystems.01042-22.7 TABLE S1 Differentially expressed genes in the *B. cinerea* Δ*bcxyr1* mutant versus the wild-type strain B05.10. Download Table S1, DOCX file, 0.02 MB. Copyright © 2022 Ma et al. 2022 Ma et al. https://creativecommons.org/licenses/by/4.0/ This content is distributed under the terms of the Creative Commons Attribution 4.0 International license.

10.1128/msystems.01042-22.8 TABLE S2 qRT-PCR quantification of the gene expression on nine selected genes. Download Table S2, DOCX file, 0.02 MB. Copyright © 2022 Ma et al. 2022 Ma et al. https://creativecommons.org/licenses/by/4.0/ This content is distributed under the terms of the Creative Commons Attribution 4.0 International license.

**FIG 5:**
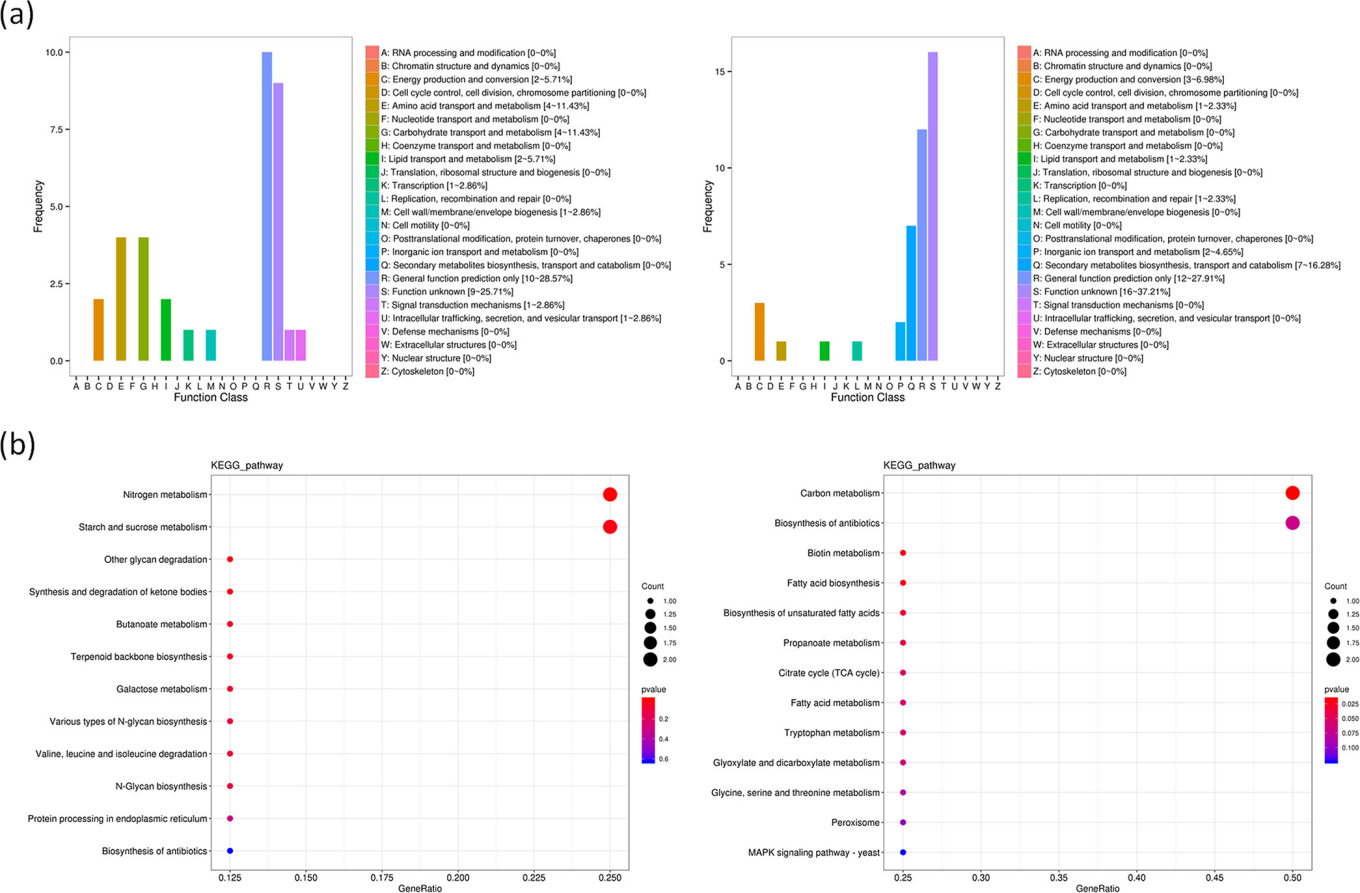
Analysis of differentially expressed genes in the Δ*bcxyr1* mutant compared the wild-type strain. (a) eggNOG function classification of downregulated genes (left) and upregulated genes (right) in the Δ*bcxyr1* strain. (b) KEGG pathways enrichment analysis of downregulated genes (left) and upregulated genes (right) in the Δ*bcxyr1* strain.

Further, the evolutionary genealogy of genes: nonsupervised orthologous groups (eggNOG) database was used to analyze the function classification of the BcXyr1-regulated genes. As shown in Fig. 5a, the main categories represented among the 41 downregulated genes in the Δ*bcxyr1* strain were those participating in carbohydrate transport and metabolism and amino acid transport and metabolism. In contrast, the 54 upregulated genes were enriched with those involved in secondary metabolite biosynthesis, transport, and catabolism. The Kyoto Encyclopedia of Genes and Genomes (KEGG) pathway analysis also indicated that deletion of *bcxyr1* repressed the expression of genes involved in carbohydrate metabolic pathways, including starch and sucrose metabolism, other glycan degradation, galactose metabolism, and N-glycan biosynthesis, and stimulated the expression of genes involved in carbon metabolism and the biosynthesis of antibiotics (Fig. 5b).

Out of the 41 genes that were downregulated in the Δ*bcxyr1* strain, 22 genes harbor a predicted signal peptide, and half of them were CAZymes, including those participating in plant cell wall degradation (see Table S3 in the supplemental material). For instance, the previously reported virulence-associated gene *bcpg2* (31) was downregulated in the Δ*bcxyr1* strain, which suggests that BcXyr1 positively regulates the expression of PCWDE genes. In addition to carbohydrate metabolism, several genes related to nitrogen transport and metabolism were downregulated, including *bccrnA* (Bcin01g06290) that encodes a putative nitrate/nitrite transporter, *bcniaD* (Bcin07g01270), which encodes nitrate reductase that is involved in the first step of nitrate assimilation (32), and a gene encoding putative amino acid permease (Bcin05g02810). Furthermore, *bcxyr1* was required for the expression of the glucose-oxidase-encoding gene *bcgod1* (Bcin14g05500) (33) and the galactose-oxidase-encoding gene *bcgox1* (Bcin13g05710), which generate H_2_O_2_ as a by-product during the oxidation of glucose or galactose, respectively. Deletion of *bcxyr1* also affected the expression of the CDIP BcNep2, which causes necrosis in dicotyledonous plant species (34). On the other side, genes upregulated in the Δ*bcxyr1* strain were enriched in transporters, particularly MFS transporters (Bcin06g06880, Bcin10g04810, Bcin09g05240, Bcin12g01880, and Bcin09g00730), and four genes (Bcin05g08400, Bcin11g02640, Bcin03g06680, and Bcin02g07640) involved in secondary metabolism were also negatively regulated by BcXyr1. Interestingly, a probable succinyl-coenzyme A (CoA)-ligase-encoding gene (Bcin11g03450) was upregulated in the Δ*bcxyr1* strain. Recently, succinate was shown to be connected to stress responses and cell death (35), suggesting this gene might play a role in the host-pathogen interaction.

10.1128/msystems.01042-22.9 TABLE S3 Secreted protein-encoding genes downregulated in the Δ*bcxyr1* strain. Download Table S3, DOCX file, 0.02 MB. Copyright © 2022 Ma et al. 2022 Ma et al. https://creativecommons.org/licenses/by/4.0/ This content is distributed under the terms of the Creative Commons Attribution 4.0 International license.

**FIG 6:**
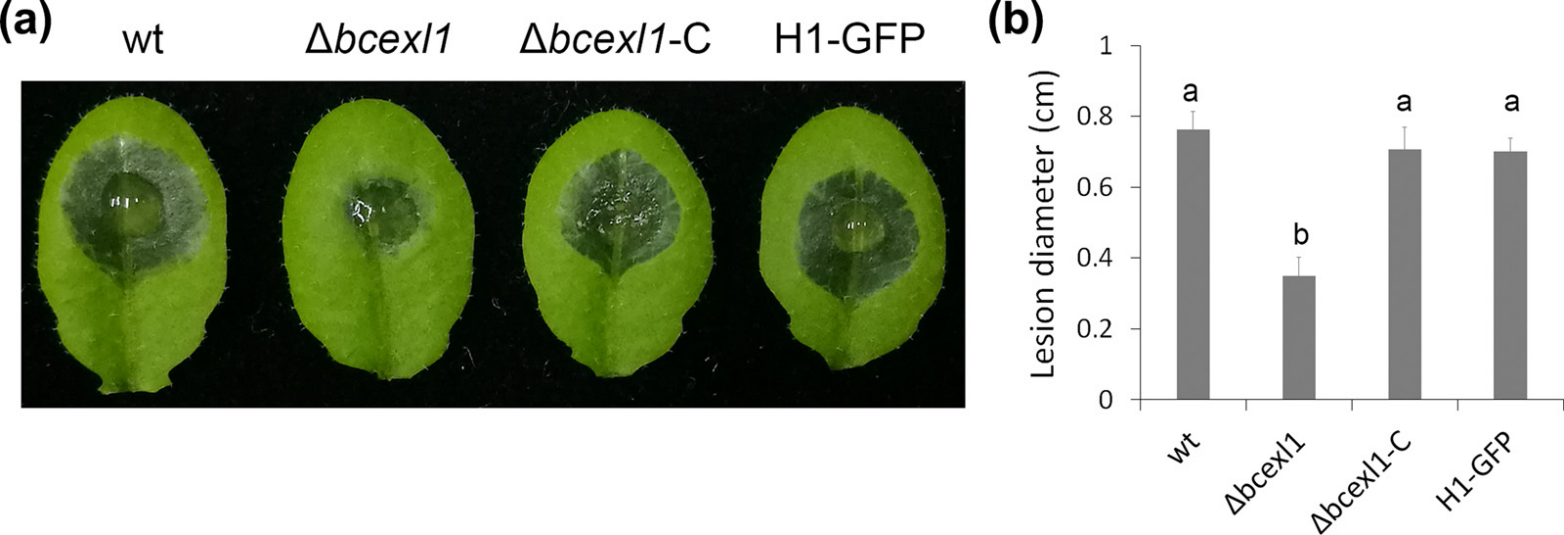
Deletion of *bcexl1* affects fungal virulence. Spores were diluted to 10^5^/mL in liquid CD + 1% sucrose medium, and 7.5 μL of spore suspension was inoculated onto *Arabidopsis* leaves. (a) Typical lesions developed at 75 hpi. (b) Average lesion size at 75 hpi. Data are the means ± SD of eight leaves for each strain. Columns marked by different letters represented statistical differences (*P < *0.01).

To investigate the function of the downregulated CAZyme genes in the *bcxyr1* deletion strain, we selected genes that encode secreted proteins, which have been detected in the secretome. Among these genes, the protein product of a putative expansin-like gene (Bcin01g02460) was found previously in the *B. cinerea* secretome (36–38), and therefore, we generated a deletion strain of this gene that we named Δ*bcexl1* and tested its pathogenicity. The Δ*bcexl1* strain was hypovirulent and caused smaller lesions than the wild-type strain (Fig. 6). Transformation of the Δ*bcexl1* strain with the native *bcexl1* gene restored full virulence, confirming that the reduced pathogenicity of Δ*bcexl1* resulted from deletion of the *bcexl1* gene. To the best of our knowledge, expansin genes have not been reported to be involved in fungal pathogenesis, and therefore, how this gene functions during the plant-pathogen interaction remains to be elucidated.

### Virulence is further reduced but not abolished in the Δ*bcexl1*Δ*bcxyr1* double deletion strain.

**FIG 7:**
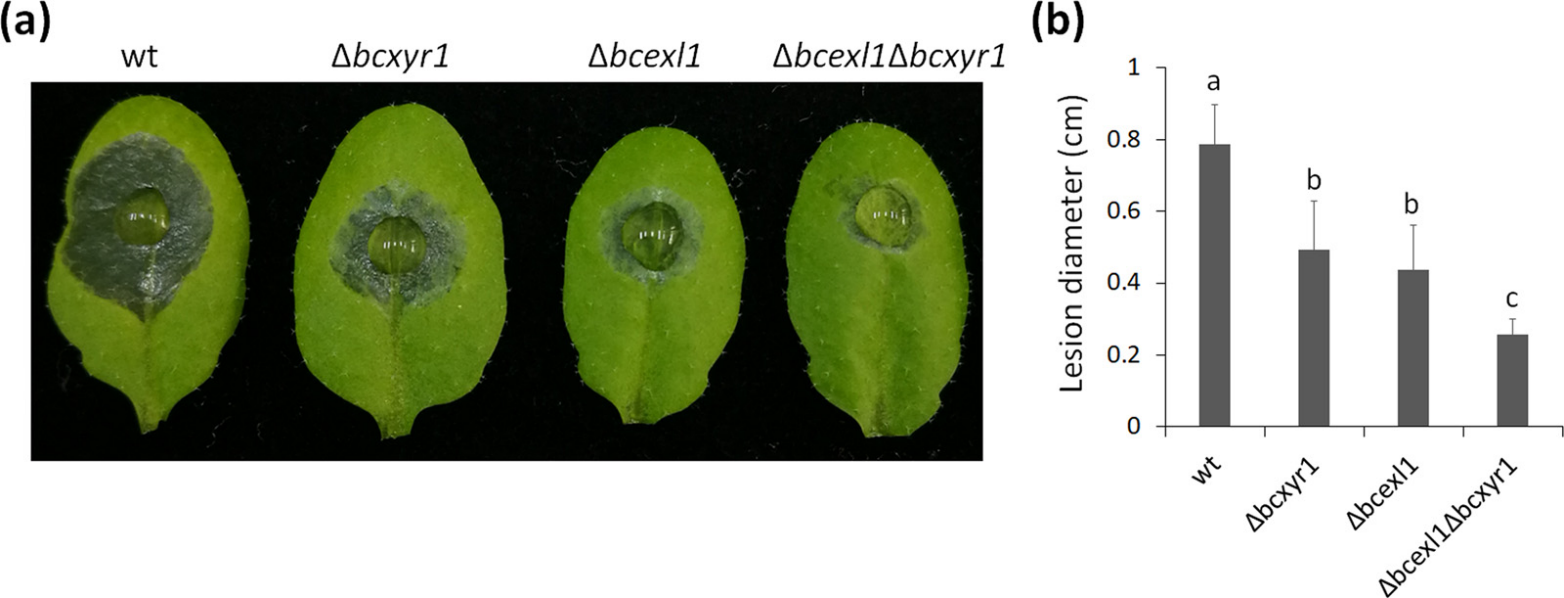
Double deletion of *bcexl1* and *bcxyr1* results in further reduction of fungal virulence. Spores were diluted to 10^5^/mL in liquid CD + 1% sucrose medium, and 7.5 μL of spore suspension was inoculated onto *Arabidopsis* leaves. (a) Typical lesions developed at 84 hpi. (b) Average lesion size at 84 hpi. Data are the means ± SD of eight leaves for each strain. Columns marked by different letters represented statistical differences (*P < *0.01).

To estimate if the effect of losing both genes is greater than that of losing a single one, we generated a Δ*bcexl1*Δ*bcxyr1* double-deletion strain by deleting the *bcxyr1* gene on a background of the Δ*bcexl1* strain. Compared with either Δ*bcexl1* or Δ*bcxyr1*, the double-deletion strain showed a weaker virulence at 84 hpi, although pathogenicity was not completely abolished (Fig. 7). These results suggest that (i) along with *bcexl1*, other genes regulated by *bcxyr1* also contribute to fungal virulence; (ii) *bcexl1* is not fully controlled by *bcxyr1*, and the remaining expression of *bcexl1* in the Δ*bcxyr1* strain still plays a role in virulence, reflecting the importance of *bcexl1* in pathogenicity; and (iii) repressing a portion of virulence-associated genes will probably be insufficient to achieve complete elimination of the fungal virulence. Therefore, *bcexl1* and *bcxyr1* play crucial but distinct roles in fungal virulence.

## DISCUSSION

PCWDEs are considered essential components of *B. cinerea* virulence arsenal; however, conclusive evidence for their role in pathogenicity is limited, possibly due to functional redundancy. In this study, we showed that the *B. cinerea* transcription factor BcXyr1 regulates (hemi-)cellulase production as well as the expression of additional PCWDEs. Deletion of *bcxyr1* resulted in reduced virulence, demonstrating the contribution of PCWDEs to fungal virulence. We further identified a BcXyr1-regulated expansin-like gene (*bcexl1*), which was required for full virulence.

The transcription factor Xyr1 (Xlr1/XlnR) is a major regulator of genes encoding xylanolytic and cellulolytic enzymes in different fungi (21). In this study, deletion of *bcxyr1* reduced the secretion of xylanases and cellulases, confirming the regulation of (hemi-)cellulase genes by BcXyr1. This result was further confirmed by RNA-seq analysis, which revealed 41 genes that were positively regulated by BcXyr1, of which 13 genes were categorized as CAZymes. Deletion of *bcxyr1* significantly affected fungal virulence (Fig. 3), in contrast to studies in other fungi, such as Magnaporthe oryzae (24), Fusarium oxysporum (23), and Fusarium graminearum (22), in which *bcxyr1* homologues were dispensable for virulence. There could be several reasons underlying the difference. First, the function of Xyr1/XlnR varies in different fungi, for example, in M. oryzae, XlnR was found to be involved in the transcriptional control of the pentose catabolic pathway but not genes encoding (hemi-)cellulolytic enzymes (24), while in F. oxysporum, XlnR controlled xylanase gene expression but did not affect cellulase activity (23). Second, although disruption of Xyr1 regulated both cellulase and xylanase production in F. graminearum, the residual PCWDEs could be sufficient to achieve infection (22). In addition, M. oryzae and most Fusarium species are hemibiotrophs and the role of XlnR/Xyr1 might differ in these classes compared with necrotrophic pathogens, such as *B. cinerea.*

A working model proposed that during the early infection phase, *B. cinerea* secretes necrosis-inducing factors (CDIPs and toxins) that produce small spots of dead tissue, in which the fungus can establish itself and use as foci for spreading in the following stages (25). The N. benthamiana infiltration test showed that the secretome of the Δ*bcxyr1* strain displayed weaker cell-death-inducing activity than that of the wild type (Fig. 3a), suggesting that BcXyr1 may regulate the expression of certain PCWDEs that are also CDIPs (17, 39).

Among the 11 BcXyr1-regulated secreted CAZyme-encoding genes (Table S3), we investigated *bcexl1* that encodes a putative expansin-like protein, and it was found essential for *B. cinerea* virulence. Unlike most other types of PCWDEs, *BcExl1* is the only expansin-like protein found in the *B. cinerea* secretome, possibly explaining the clear phenotype of the Δ*bcexl1* strain. Therefore, the impaired virulence of the Δ*bcxyr1* strain is at least partially due to the reduced expression of *bcexl1* and possibly other PCWDEs, which also correlates with the observation that the secretome of the Δ*bcxyr1* strain displayed a weaker cell-death-inducing ability. Expansins are proteins that are present primarily in the plant kingdom and are known to have cell-wall-loosening activity and to be involved in cell expansion and other developmental events (40). So far, expansin-like proteins have hardly been investigated in plant-pathogenic fungi, and it would be interesting and important to gain insight into the role of *bcexl1* in the pathogen-plant interaction.

The development of CRISPR-Cas genome editing technology in *B. cinerea* enables the generation of multiple knockout mutants (17) and is expected to facilitate the evaluation of multiple genes at one time. Application of CRISPR-Cas will make it much easier to identify those virulence-associated ones from numerous downstream genes.

The CAZymes that are produced by plant-pathogenic fungi play a pivotal role in breaching the frontline of plant defense (5). Biotrophs, hemibiotrophs, and necrotrophs vary not only in the number of CAZyme genes but also in the expression pattern of CAZyme genes. For example, most CAZyme genes of the necrotroph *B. cinerea* were expressed during infections of lettuce leaves, ripe tomato fruit, and grape berries (8), while many CAZymes are not expressed in the biotroph Cladosporium fulvum during plant infection (41). Since Xyr1 (Xlr1/XlnR) is considered a major regulator of fungal cellulase and hemicellulose genes, we anticipate that a comparative analysis of the regulon of BcXyr1 and its homologues in pathogens of different lifestyles would contribute to better understanding the necrotrophic infection mechanisms of *B. cinerea*. Taken together, in this study, we identified the crucial PCWDE-regulating transcription factor BcXyr1 that regulates both (hemi-)cellulase production and fungal virulence in *B. cinerea*, providing evidence that supports the role of PCWDEs in the pathogenicity of *B. cinerea*.

## MATERIALS AND METHODS

### Fungi and plants.

Botrytis cinerea strain B05.10 was used as the wild-type strain. Potato dextrose agar (PDA; Acumedia, Lansing, MI) was used as maintenance and sporulation medium. Routine culture or growth assays were carried out in the following media: Gamborg B5 including vitamin mixture (GB5; Duchefa Biochemie) supplemented with the indicated carbon sources and Czapek Dox medium (CD; 0.3% NaNO_3_, 0.05% KCl, 0.05% MgSO_4_·7H_2_O, 0.001% FeSO_4_·7H_2_O, 0.1% K_2_HPO_4_·3H_2_O [pH 7.3]) supplemented with the indicated carbon sources. Solid media were prepared by adding 1.5% agar.

Arabidopsis thaliana ecotype Columbia (Col-0) plants were grown in a 14-/10-h light/dark cycle at 20°C. Tomato plants and cucumber plants were grown in a 16-/8-h light/dark cycle at 25°C. Mature leaves plants were used for infection assays. The collection of spores and infection procedures were performed as described previously (42).

### Generation of *B. cinerea* mutant strains.

The *B. cinerea* B05.10 genome sequence was used to design primers (http://fungi.ensembl.org/Botrytis_cinerea/Info/Index). Name, purpose, and sequences of all the primers are listed in Table S4 in the supplemental material. Single-deletion mutants (Δ*bcxyr1*) were generated by replacing the entire open reading frame (ORF) of *bcxyr1* with a hygromycin resistance cassette (*hph*) (Fig. S1a). The *hph* cassette consists of the Aspergillus nidulans *oliC* promoter (*PoliC*), the hygromycin phosphotransferase gene (*hph*), and *acr* (*B. cinerea* enoyl- [acyl carrier protein] reductase) terminator (*Tacr*) (27). A similar tactic was applied to generate the *bcexl1* deletion strain (see Fig. S5 in the supplemental material).

10.1128/msystems.01042-22.5 FIG S5 Generation of the Δ*bcexl1* strain. The deletion cassette contained the hygromycin resistance gene (*hph*) flanked by the upstream region and the downstream region of the *bcexl1* gene. The primers used to verify the transformants are indicated (arrows). Download FIG S5, TIF file, 0.2 MB. Copyright © 2022 Ma et al. 2022 Ma et al. https://creativecommons.org/licenses/by/4.0/ This content is distributed under the terms of the Creative Commons Attribution 4.0 International license.

10.1128/msystems.01042-22.10 TABLE S4 Primers used in this study. Download Table S4, DOCX file, 0.02 MB. Copyright © 2022 Ma et al. 2022 Ma et al. https://creativecommons.org/licenses/by/4.0/ This content is distributed under the terms of the Creative Commons Attribution 4.0 International license.

Constructs for the deletion of the *bcxyr1* gene were produced by adding 500 bp of the 5′ flanking region of the desired gene on one side of the *hph* cassette and 500 bp of the 3′ flanking region on the other side by overlap PCR. A *bcxyr1* complementation construct was prepared by assembling four PCR fragments using a Gibson assembly master mix kit (New England BioLabs [NEB]). The complementation cassette contained the upstream region of *bcxyr1* and the *bcxyr1* ORF, the *Tcel5a* termination signal (*B. cinerea cel5a*, GenBank accession number AY618929.1), a *nr* cassette that conferring nourseothricin resistance, and the downstream region of *bcxyr1* (Fig. S1b).

A *bcxyr1* overexpression cassette was prepared by placing the *bcxyr1* ORF under the *B. cinerea* strong H2B promoter (27) and was flanked by a *hph* resistance gene (Fig. S1c). A fragment containing the 3′ part of the *bcgapdh* ORF (Bcin15g02120) and its termination sequence and a fragment of 600 bp adjacent to the *bcgapdh* termination sequence were then placed upstream and downstream of the *PH2B-bcxyr1*-*hph* cassette, respectively. The cassette was then inserted to the *bcgapdh* locus by homologous recombination.

To generate a *bcexl1* complementation strain, an intact *bcexl1* fragment (2,070 bp) containing 500 bp of the 5′ flanking sequence and 500 bp of the 3′ flanking sequence was amplified using genomic DNA as the template. The H1-green fluorescent protein (GFP) cassette (see Fig. S6 in the supplemental material) that was designed to tag the nuclei with GFP (25) was constructed by fusing the 3′ part of the histone H1 gene (Bcin02g04870), GFP, the *Tcel5a* termination signal, the *nr* gene, and the 3′ flanking region of the histone H1 gene together. The *bcexl1* fragment and the H1-GFP cassette were cotransformed into the Δ*bcexl1* strain to generate the complementation strain. The H1-GFP cassette was transformed into wild-type strain to generate the H1-GFP strain.

10.1128/msystems.01042-22.6 FIG S6 Generation of a *bcexl1* complementation strain by cotransformation of the Δ*bcexl1* strain with the native *bcexl1* fragment and H1-GFP cassette. Download FIG S6, TIF file, 0.4 MB. Copyright © 2022 Ma et al. 2022 Ma et al. https://creativecommons.org/licenses/by/4.0/ This content is distributed under the terms of the Creative Commons Attribution 4.0 International license.

A double-deletion mutant (Δ*bcexl1*Δ*bcxyr1*) was generated by replacing the *bcxyr1* ORF with *nr* gene on the background of the Δ*bcexl1* strain. The *bcxyr1* deletion cassette was constructed by adding 500 bp of the 5′ flanking region of *bcxyr1* on one side of the *nr* gene and 500 bp of the 3′ flanking region on the other side by overlap PCR.

Genetic transformation of *B. cinerea* with the different DNA constructs was performed as described previously (27). Colonies that developed on the selection media were transferred to separate plates. At least 10 independent transformants for each mutant were obtained for each mutant, and genomic DNA was extracted from each colony and analyzed by PCR to verify the integration of the construct at the desired locus. Homokaryotic strains were obtained by single-spore isolation, and derived colonies were analyzed by PCR to verify that the strain is homokaryotic at the desired locus and additional rounds of single-spore isolation were performed in cases of impurity. At least four separate single-spore isolates were obtained for each strain, and similar experimental results were observed from independent transformants. In the initial analyses, we used *bcxyr1* deletion strain Δ*bcxyr1*-1a1 (abbreviated Δ*bcxyr1*), *bcxyr1* complementation strain Δ*bcxyr1*-C1b (abbreviated Δ*bcxyr1*-C), *bcxyr1* overexpression strain ox-*bcxyr1*-6b (abbreviated ox-*bcxyr1*), *bcexl1* deletion strain Δ*bcexl1*-9a1 (abbreviated Δ*bcexl1*), *bcexl1* complementation strain Δ*bcexl1*-C2i (abbreviated Δ*bcexl1*-C), *bcexl1* and *bcxyr1* double-deletion strain Δ*bcexl1*Δ*bcxyr1-3c* (abbreviated Δ*bcexl1*Δ*bcxyr1*), and H1-GFP homologous transformant H1-GFP-7a (abbreviated H1-GFP).

### (Hemi-)cellulase assay.

Spores were collected from 1-week-old PDA cultures as described previously (43). To compare (hemi-)cellulase production, 5 μL of spore suspensions (10^5^/mL) was inoculated into 1/2 Gamborg B5 solid medium supplemented with 0.5% carboxymethyl cellulose (241297; J&K Chemical Ltd., China) or 0.2% beechwood xylan (X4252; Sigma, Germany). After 4 days of fungal growth, a 0.1% Congo red solution and a 1 M NaCl solution was used to flood the plate sequentially, and the halo diameters of different strains were measured.

To further measure the (hemi-)cellulase activity of different strains, spore suspensions were added into the liquid malt medium in Erlenmeyer flasks to a final concentration of 10^6^/mL. Samples were incubated on an orbital shaker with agitation at 150 rpm for 20 h at 21°C. Then samples were centrifuged at 4,000 × *g* for 10 min, and the supernatant was removed. Mycelium pellets were washed with sterile deionized distilled water (DDW) and then centrifuged to decant the supernatant, and this washing cycle was repeated three times to get rid of the residual malt medium. Then 0.3 g of wet mycelial sample was inoculated into a 50-mL Erlenmeyer flask containing 10 mL of Gamborg B5 liquid medium supplemented with 2% rice straw powder as a (hemi-)cellulase inducer, and samples were incubated with agitation at 150 rpm for 24 h. Then samples were collected and centrifuged at 12,000 × *g* for 20 min, and the supernatants were filtered through 0.22-μm filters and subjected to (hemi-)cellulase activity measurement. Cellulase activity was determined as follows: 25-μL supernatant was incubated with a 25-μL CMC solution (2% in 0.05 M acetate buffer, pH 5.0) at 30°C for 30 min. Then 100 μL of dinitrosalicylic acid (DNS) solution was added and the mixtures were incubated at 95°C for 5 min. The absorbance at 540 nm was measured with a Multiskan GO microplate spectrophotometer (Thermo Scientific). Xylanase activity was determined as follows: 25-μL supernatant was incubated with 25-μL beechwood xylan solution (1% in 0.05 M acetate buffer, pH 5.0) at 30°C for 30 min. Then 100 μL of DNS solution was added and the mixtures were incubated at 95°C for 5 min. One international unit (IU) of the activity of cellulase or xylanase was defined as the amount of enzyme to liberate one micromole (μM) reducing sugars per minute from CMC or beechwood xylan.

### Pathogenicity test.

Pathogenicity assays were performed on *Arabidopsis*, tomato, and cucumber plants as described previously with modifications (27). Briefly, leaves were inoculated with 7.5-μL droplets of spore suspension (10^5^/mL in liquid CD + 1% sucrose medium) or with mycelial plugs (3 mm in diameter) from a CD + 1% sucrose agar plate.

To test the induction of plant cell death by secreted proteins of different strains, the culture supernatants from strains which grew 24 h in Gamborg medium plus 2% rice straw powder were collected, filtered, and concentrated using 3-kDa MWCO Amicon Ultra-15 centrifugal filter units (Millipore) at 4°C. Then the concentrated proteins were diluted in PBS to a final concentration of 20 μg/mL and infiltrated into N. benthamiana leaves using syringes. Plants were kept in a growth chamber at 25°C for 5 days.

### RNA isolation, sequencing, and data analysis.

Spore suspensions of *B. cinerea* wild-type strain 05.10 and Δ*bcxyr1* strain were added into liquid malt medium in Erlenmeyer flasks to a final concentration of 10^6^/mL, and three biological replicates were used for each sample. Samples were incubated on an orbital shaker with agitation at 150 rpm for 20 h at 21°C. Then samples were centrifuged at 4,000 × *g* for 10 min, and the supernatants were removed. Mycelial pellets were washed with sterile DDW and then centrifuged to decant the supernatant. This washing cycle was repeated five times to thoroughly get rid of the residual malt medium. For each sample, 0.5 g of wet mycelia was inoculated into a 50-mL Erlenmeyer flask containing 10 mL liquid CD + 1% sucrose medium and incubated with agitation at 150 rpm for 7 h. Then mycelia of each sample were harvested and ground under liquid nitrogen to extract total RNA using the RNAprep pure plant kit (TianGen, China).

A total amount of 1 μg RNA per sample was used as input material for the RNA sample preparations. Sequencing libraries were generated using NEBNext Ultra RNA library prep kit for Illumina (NEB, USA) following manufacturer’s recommendations, and index codes were added to attribute sequences to each sample. The clustering of the index-coded samples was performed on a cBot cluster generation system using TruSeq PE cluster kit v4-cBot-HS (Illumina) according to the manufacturer’s instructions. After cluster generation, the library preparations were sequenced on an Illumina platform and paired-end reads were generated.

Raw data (raw reads) of FASTQ format were first processed through in-house Perl scripts. In this step, the adaptor sequences and low-quality sequence reads were removed from the data sets. At the same time, Q20, Q30, GC content, and sequence duplication level of the clean data were calculated. These clean reads were then mapped to the *B. cinerea* 05.10 genome using the HISAT2 program (44). Only reads with a perfect match or one mismatch were further analyzed and annotated based on the reference genome. Gene expression levels were estimated by the number of fragments per kilobase per million fragments mapped (FPKM). Differential expression values were determined using DEseq (45), and the false discovery rate (FDR) of <0.05 and fold change of ≥2 were set as the thresholds for significantly differential expression.

### Real-time quantitative PCR (qRT-PCR).

The RNA samples were reverse transcribed into cDNA using PrimeScript RT reagent kit with genomic DNA (gDNA) Eraser (TaKaRa, Dalian, China) according to the manufacturer’s protocol. For the reaction, SYBR Premix *Ex Taq* II (TaKaRa, Dalian, China) was used for 20-μL assays. Primers used were given in Table S4. Three replicates were performed per experiment. The *bcgpdh* gene (43) was used as a control gene to normalize data. qRT-PCR was performed using the CFX Connect real-time system (Bio-Rad). The experiments were repeated three times. Nine genes were chosen to validate the RNA-seq results using the qRT-PCR method.

### Spore germination assay.

Spore suspension from each sample was prepared in liquid CD + 1% sucrose medium to a concentration of 10^5^/mL. A droplet of 15 μL was pipetted onto a microscope cover glass (0101050; Marienfeld, Germany), and kept in a humid chamber. Besides, 7.5 μL of spore suspension was pipetted onto *Arabidopsis* leaves and kept in a humid box. Spore germination was then examined using microscopes.

### Lactophenol trypan blue staining.

Infected leaves were stained with lactophenol trypan blue as described previously (27). The *Arabidopsis* leaf tissue around the inoculated spot was cut and transferred into tubes containing 2 mL of fresh trypan blue staining solution (10 mL of lactic acid, 10 mL of glycerol, 10 mL of phenol [saturated with Tris-buffer, pH 8], and 10 mg of Trypan blue, mixed well with 10 mL of sterile DDW). The tubes were boiled for 2 min, and samples were then distained for 1 h in chloral hydrate solution (2.5 g chloral hydrate in 1 mL DDW). Stained samples were mounted on microscope slides and examined under an SMZ25 stereomicroscope (Nikon, Japan).

### Hydrogen peroxide detection.

*Arabidopsis* leaves were inoculated with 7.5-μL droplets of spore suspension (10^5^/mL in liquid CD + 1% sucrose medium). After 24 h and 48 h, inoculated leaves were stained with 0.1% 3, 3′-diaminobenzidine (DAB) staining solution to detect H_2_O_2_ accumulation as described (46). Pictures were taken using a Nikon SMZ25 stereomicroscope. The brown intensity of DAB staining was quantified using ImageJ software (https://imagej.nih.gov/ij/).

### Statistical analyses.

The statistical significance tests were performed by Student’s *t* test (*, *P* < 0.01; two-tailed test). In all graphs, results represent the mean value of at least three independent experiments, each with at least three replications per treatment.

### Data availability.

Raw RNA-seq data can be accessed at the SRA database (BioProject identifier [ID] PRJNA762449).
