# Supplementary figures and images for: Botrytis cinerea Transcription Factor BcXyr1 Regulates (Hemi-)Cellulase Production and Fungal Virulence
Source: mSystems. 2022 Dec 5;7(6):e01042-22. doi: 10.1128/msystems.01042-22 (PMC9765177; doi:10.1128/msystems.01042-22)

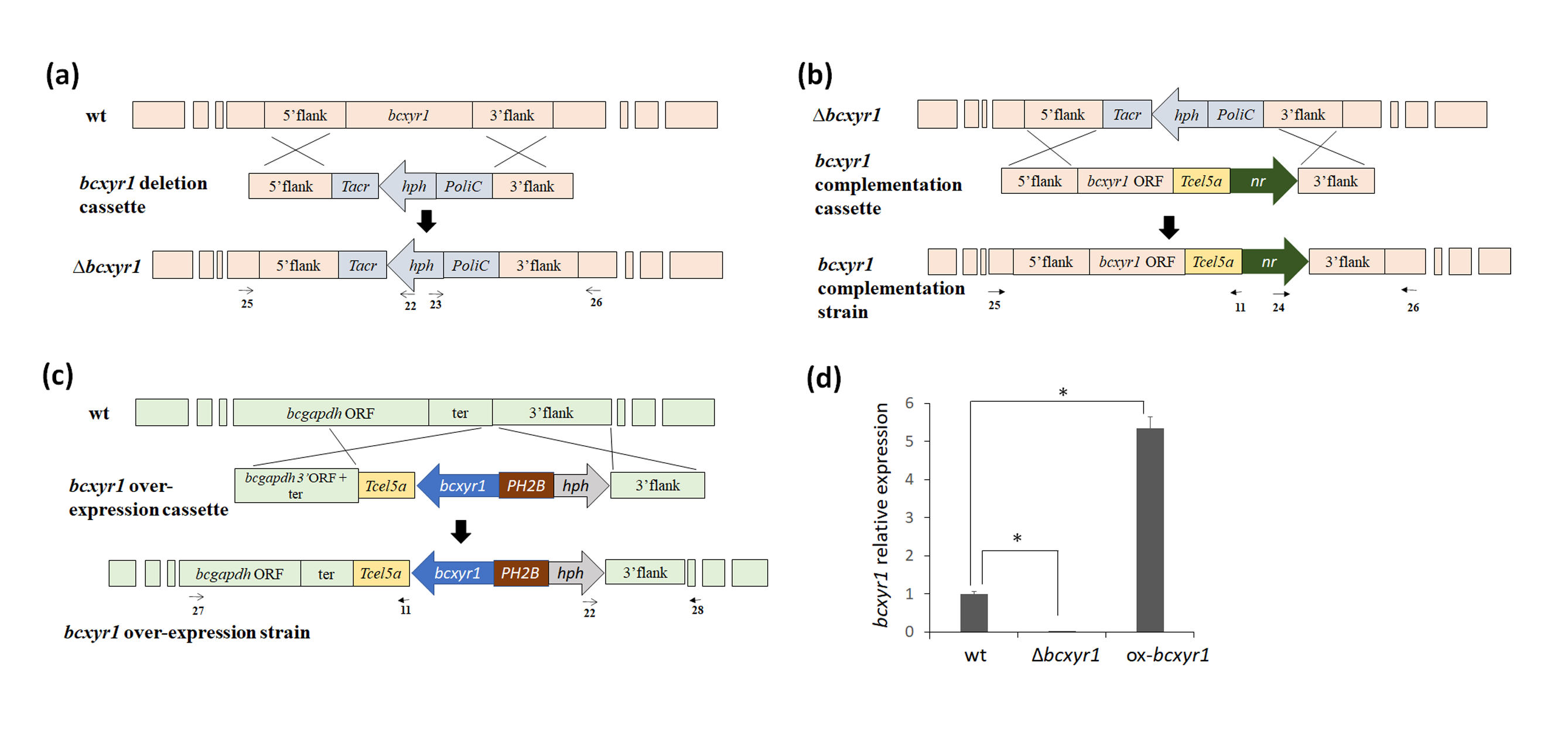

Supplement: FIG S1 [file msystems.01042-22-s0001.tif]

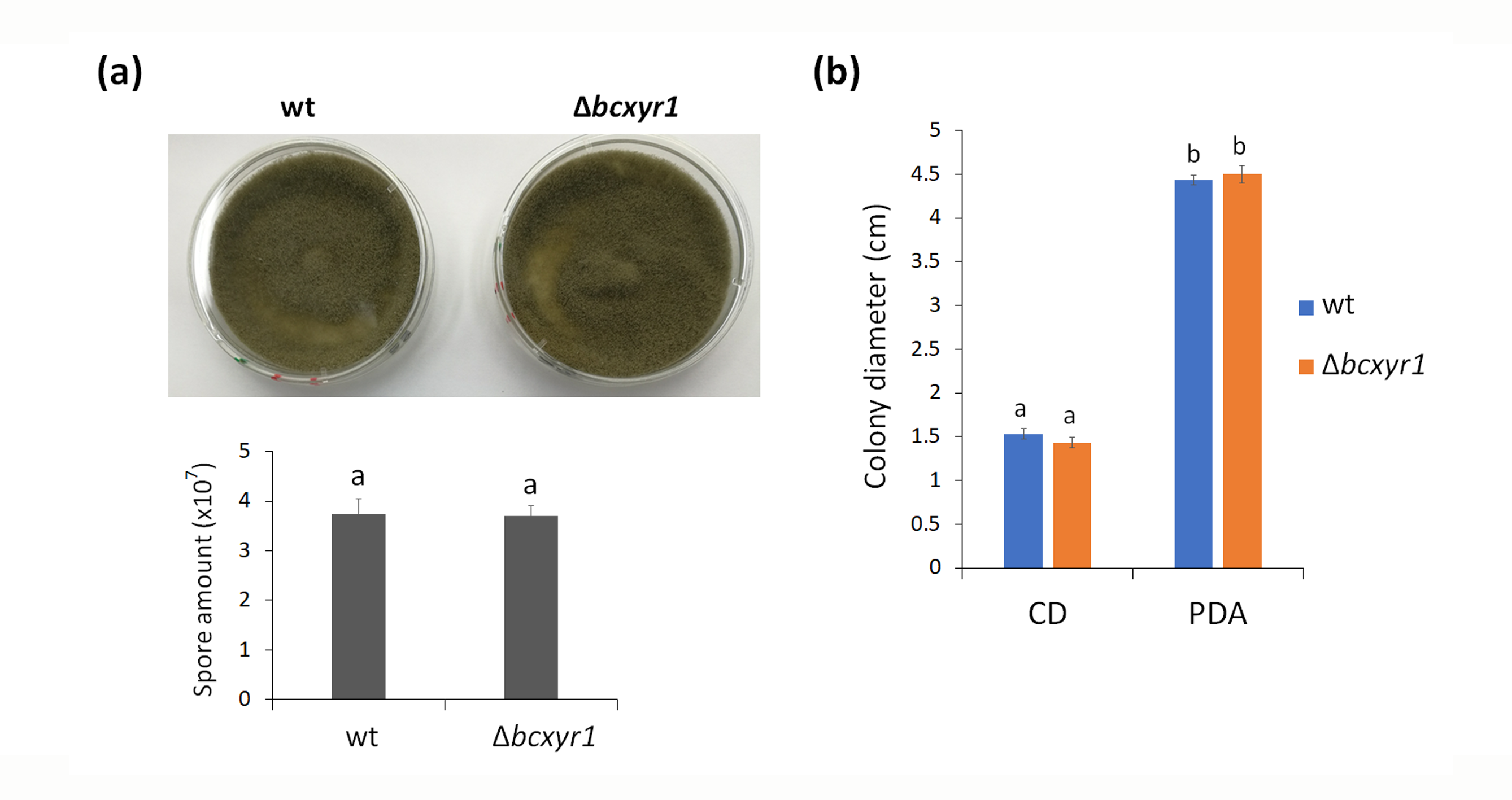

Supplement: FIG S2 [file msystems.01042-22-s0002.tif]

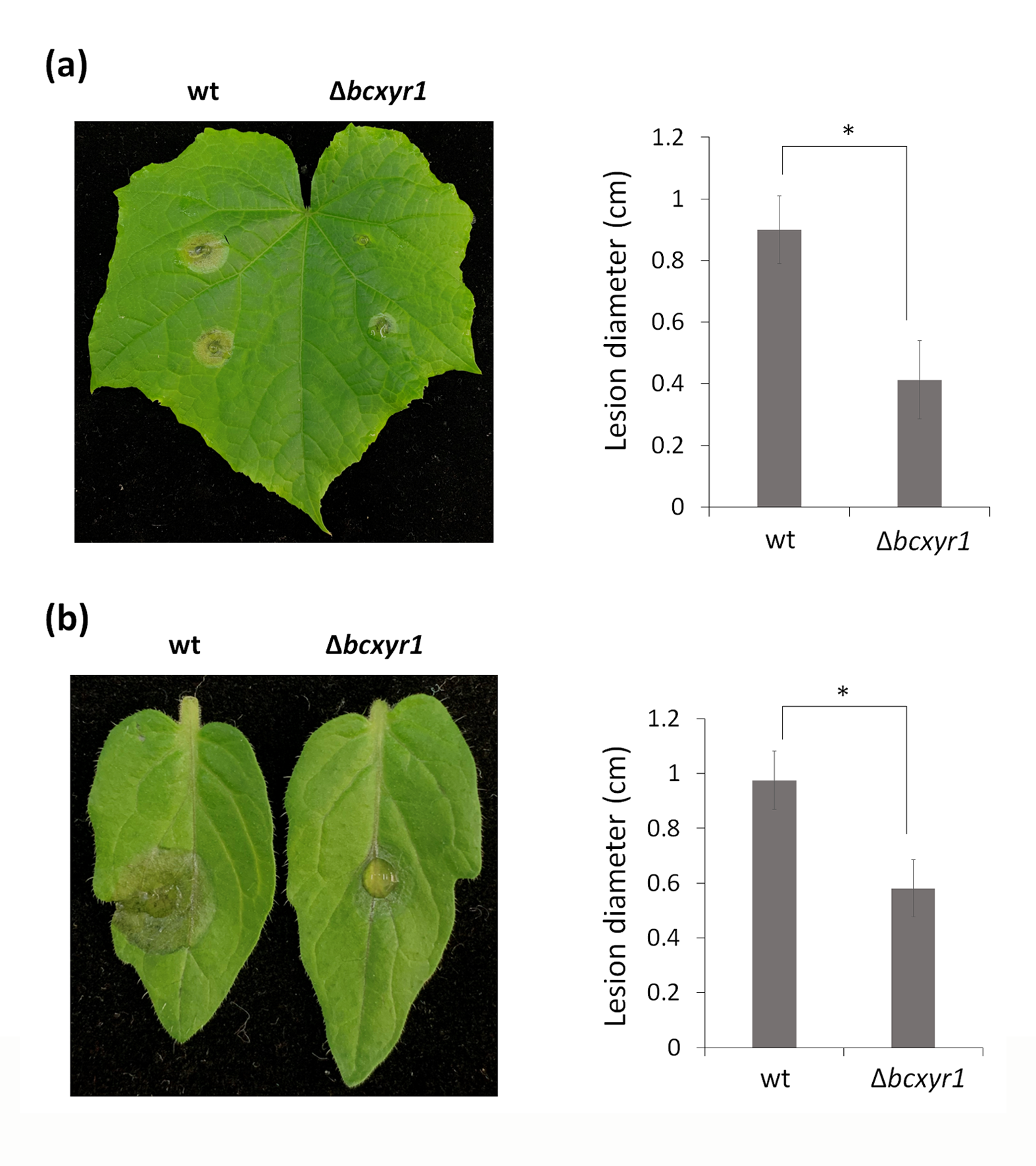

Supplement: FIG S3 [file msystems.01042-22-s0003.tif]

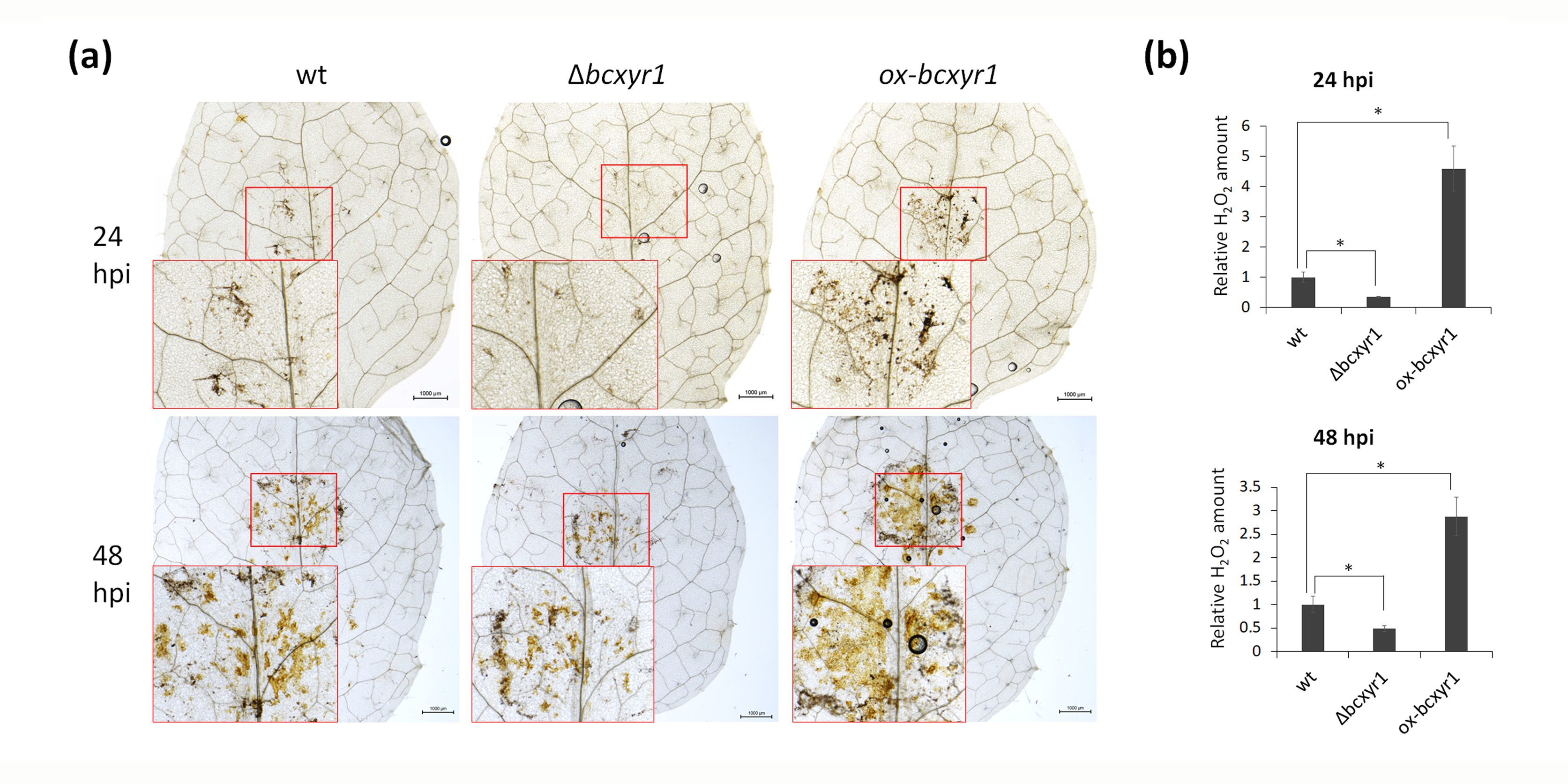

Supplement: FIG S4 [file msystems.01042-22-s0004.tif]

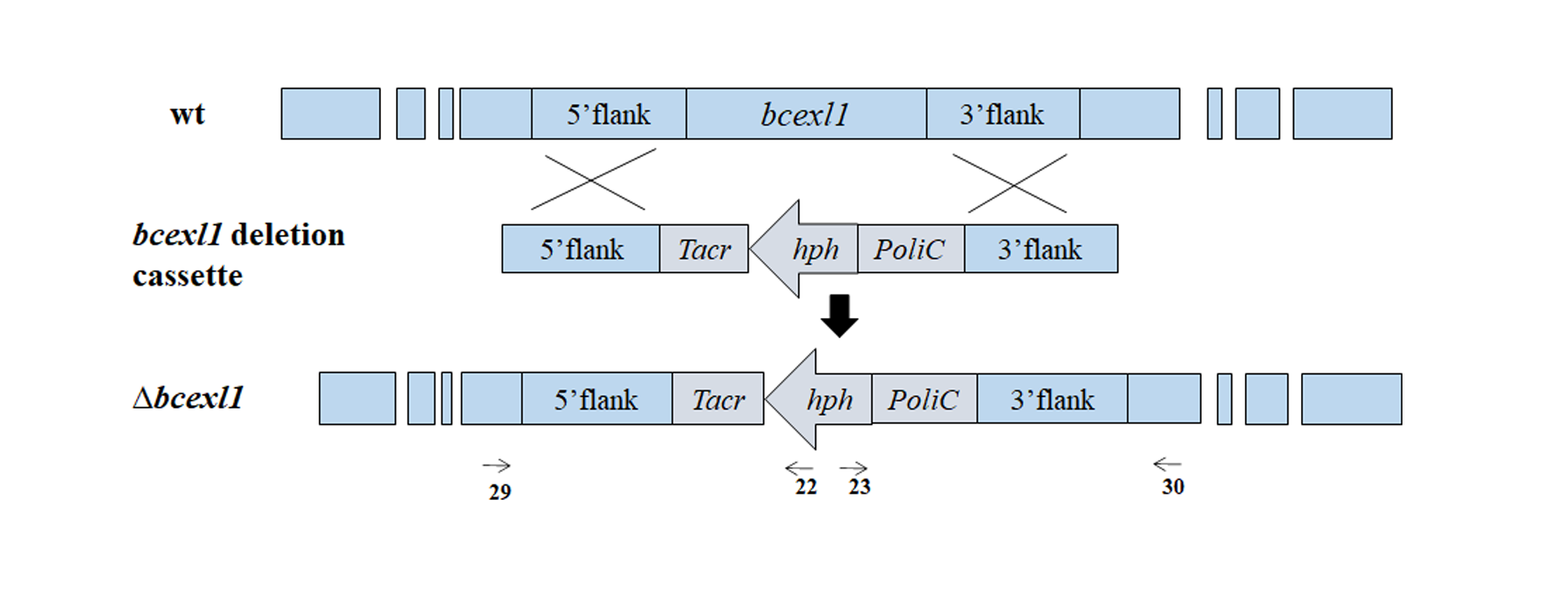

Supplement: FIG S5 [file msystems.01042-22-s0005.tif]

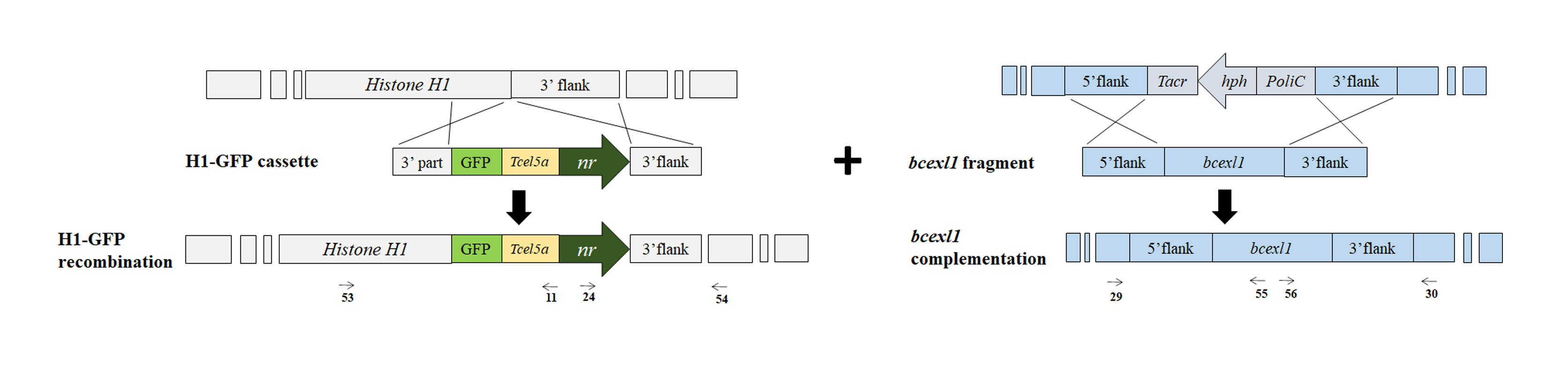

Supplement: FIG S6 [file msystems.01042-22-s0006.tif]
