# Supplementary material for: Botrytis cinerea Transcription Factor BcXyr1 Regulates (Hemi-)Cellulase Production and Fungal Virulence
Source: mSystems. 2022 Dec 5;7(6):e01042-22. doi: 10.1128/msystems.01042-22 (PMC9765177; doi:10.1128/msystems.01042-22)
Supplement: TABLE S1 [file msystems.01042-22-s0007.docx]

Table S1. Differentially expressed genes in the *B. cinerea* Δ*bcxyr1* mutant versus the wild type strain B05.10.

| Gene ID | Gene Name | FDR | log2FC  (Δ*bcxyr1*: wt) | Description |
| --- | --- | --- | --- | --- |
| Bcin02g02000 |  | 5.36E-20 | -4.191 | Glycosyl hydrolases family 43 |
| Bcin14g00610 | *bcpg2* | 6.01E-85 | -3.495 | Glycosyl hydrolases family 28 |
| Bcin03g01250 |  | 5.56E-09 | -2.545 | Unknown function |
| Bcin07g02730 |  | 7.12E-06 | -2.507 | Pectate lyase, GH55 |
| NewGene_187 |  | 2.57E-14 | -2.400 | Unknown function |
| Bcin08g02110 |  | 8.18E-20 | -1.975 | Endoglucanase, GH5 |
| Bcin02g07770 | *bcnep2* | 0.000283236 | -1.880 | Necrosis inducing protein |
| Bcin02g07070 |  | 1.35E-23 | -1.825 | Unknown function |
| Bcin02g07470 |  | 8.60E-07 | -1.800 | Unknown function |
| Bcin06g05050 |  | 3.57E-12 | -1.776 | Glucoamylase, CBM20 |
| Bcin04g04450 |  | 1.22E-18 | -1.768 | Hydroxymethylglutaryl-CoA synthase |
| Bcin01g09540 |  | 4.74E-24 | -1.719 | Unknown function |
| Bcin11g02900 |  | 5.10E-14 | -1.711 | Trypsin |
| Bcin14g05500 | *bcgod1* | 1.65E-22 | -1.630 | Glucose oxidase |
| Bcin11g00630 |  | 5.67E-07 | -1.611 | Transcription factor |
| Bcin08g01800 |  | 3.62E-11 | -1.541 | Pyridoxal-dependent decarboxylase |
| Bcin08g05870 |  | 0.00032527 | -1.458 | Alpha-fucosidase A, GH65 |
| Bcin03g08130 |  | 0.029026831 | -1.401 | Dioxygenase |
| Bcin05g03680 |  | 0.042159858 | -1.383 | Unknown function |
| Bcin15g04770 |  | 3.32E-13 | -1.383 | Unknown function |
| Bcin12g02910 |  | 1.64E-18 | -1.365 | Cellobiose dehydrogenase |
| Bcin05g03190 |  | 0.000177886 | -1.354 | Unknown function |
| Bcin09g03170 |  | 0.000864178 | -1.319 | Unknown function |
| Bcin08g00290 | *bcpks19* | 0.004986301 | -1.296 | Putative polyketide synthase protein |
| Bcin01g05970 |  | 2.09E-05 | -1.212 | Transferase |
| Bcin01g06290 | *bccrnA* | 0.004629022 | -1.191 | MFS transporter |
| Bcin10g01020 |  | 5.86E-14 | -1.188 | Unknown function |
| Bcin03g08770 |  | 0.026424213 | -1.182 | Unknown function |
| Bcin06g03790 |  | 4.43E-06 | -1.171 | Glycosyl transferase family group 2 |
| Bcin08g05450 |  | 1.24E-05 | -1.162 | Unknown function |
| Bcin05g02800 |  | 7.93E-06 | -1.131 | Unknown function |
| Bcin06g03800 |  | 2.17E-13 | -1.130 | Glycosyl hydrolases family 16 |
| Bcin13g05710 | *bcgox1* | 0.000182121 | -1.116 | Galactose oxidase |
| Bcin02g04330 |  | 1.24E-12 | -1.099 | Unknown function |
| Bcin05g02810 |  | 0.004132797 | -1.086 | Amino acid transporter |
| Bcin13g05720 | *bcprd1* | 9.44E-06 | -1.085 | Dyp-type peroxidase |
| Bcin01g02460 |  | 0.026738236 | -1.079 | Putative expansin-like protein |
| Bcin15g04760 |  | 0.003007961 | -1.040 | Transferase |
| Bcin01g05680 |  | 1.85E-05 | -1.031 | Glycosyl hydrolase family 47 |
| Bcin07g00560 |  | 0.003181693 | -1.024 | Oxidoreductase |
| Bcin07g01270 | *bcniaD* | 0.000454923 | -1.013 | Nitrate reductase |
| Bcin04g05270 |  | 0.001089034 | 1.004 | Unknown function |
| Bcin11g03450 | *bclsc1* | 0.000106927 | 1.016 | Probable succinyl-CoA ligase |
| Bcin16g03320 |  | 6.38E-05 | 1.017 | Unknown function |
| Bcin14g01490 |  | 0.015921276 | 1.025 | Unknown function |
| Bcin01g07530 |  | 0.018685029 | 1.030 | Unknown function |
| Bcin07g00300 |  | 0.028160458 | 1.032 | hemerythrin domain-containing protein |
| Bcin03g00010 |  | 6.38E-05 | 1.034 | Unknown function |
| Bcin06g06880 | *bcamf1* | 7.33E-05 | 1.062 | MFS transporter |
| newGene_758 |  | 0.000408165 | 1.069 | Unknown function |
| Bcin01g02870 |  | 0.000244713 | 1.085 | Oxidoreductase |
| Bcin06g03510 |  | 0.018900407 | 1.098 | Putative transcription factor |
| Bcin05g08400 | *bcpks21* | 1.35E-08 | 1.098 | Putative beta-ketoacyl synthase |
| Bcin08g03490 | *bcldb19* | 0.000110614 | 1.103 | Putative arrestin N-terminal domain protein |
| Bcin09g02300 |  | 0.005777278 | 1.109 | RNA 2'-phosphotransferase |
| NewGene_1510 |  | 0.000796321 | 1.116 | Unknown function |
| Bcin02g03240 |  | 1.94E-05 | 1.122 | Transcription factor |
| Bcin04g05560 |  | 0.004035901 | 1.129 | Unknown function |
| Bcin14g00740 |  | 1.76E-13 | 1.150 | Unknown function |
| Bcin11g02640 |  | 0.014434851 | 1.190 | Oxidoreductase |
| Bcin11g05390 |  | 0.024255546 | 1.209 | Unknown function |
| Bcin01g07410 |  | 1.55E-06 | 1.222 | Unknown function |
| NewGene_770 |  | 0.000154587 | 1.232 | Unknown function |
| Bcin12g02110 |  | 6.57E-15 | 1.236 | Unknown function |
| Bcin04g01410 |  | 0.049482428 | 1.237 | Unknown function |
| Bcin12g03040 |  | 0.023658733 | 1.249 | Unknown function |
| Bcin15g00140 |  | 3.72E-13 | 1.281 | Putative fumarate reductase |
| Bcin05g08050 |  | 0.031557481 | 1.283 | Proline dehydrogenase |
| Bcin10g05910 |  | 0.000106933 | 1.285 | Unknown function |
| Bcin04g06930 | *bclot6* | 0.044606808 | 1.288 | NADPH-dependent FMN reductase |
| newGene_1272 |  | 0.011512604 | 1.314 | Unknown function |
| Bcin02g07630 |  | 1.98E-09 | 1.320 | Unknown function |
| Bcin03g00005 |  | 5.51E-20 | 1.321 | Aegerolysin |
| Bcin05g05450 |  | 0.020717616 | 1.328 | Enoyl-(Acyl carrier protein) reductase |
| Bcin13g00880 |  | 0.000417053 | 1.457 | Unknown function |
| Bcin09g04400 | *bccat7* | 0.003106241 | 1.493 | Catalase |
| Bcin01g06660 |  | 0.003878113 | 1.502 | Unknown function |
| Bcin11g01040 |  | 0.02259511 | 1.518 | Unknown function |
| Bcin03g06680 |  | 2.19E-05 | 1.539 | Cytochrome P450 |
| Bcin07g02370 |  | 0.00219961 | 1.574 | Putative amidotransferase |
| Bcin10g04810 |  | 0.003106241 | 1.647 | MFS transporter |
| Bcin03g01570 |  | 0.026246387 | 1.657 | Unknown function |
| Bcin02g07640 | *bclcc7* | 1.19E-08 | 1.697 | Multicopper oxidase |
| Bcin09g05240 |  | 0.003067645 | 1.747 | MFS transporter |
| Bcin06g01620 |  | 0.04754409 | 1.765 | Unknown function |
| Bcin12g01880 |  | 0.001145677 | 1.774 | MFS transporter |
| Bcin01g08120 |  | 0.014063901 | 1.807 | Unknown function |
| Bcin01g09630 |  | 5.80E-08 | 1.978 | Unknown function |
| Bcin03g01540 |  | 2.54E-13 | 2.216 | Oxidoreductase |
| Bcin12g06010 |  | 5.27E-17 | 2.328 | Fungal specific transcription factor domain |
| NewGene_800 |  | 1.08E-14 | 2.349 | Unknown function |
| Bcin13g02110 |  | 2.17E-36 | 2.380 | Oxidoreductase |
| Bcin12g02050 |  | 2.42E-20 | 2.501 | Oxidoreductase |
| Bcin09g00730 |  | 0.000118463 | 3.069 | MFS transporter |
| Bcin01g07100 | *bcctr1* | 0.040998757 | 3.286 | Copper transporter |
