# Supplementary material for: Botrytis cinerea Transcription Factor BcXyr1 Regulates (Hemi-)Cellulase Production and Fungal Virulence
Source: mSystems. 2022 Dec 5;7(6):e01042-22. doi: 10.1128/msystems.01042-22 (PMC9765177; doi:10.1128/msystems.01042-22)
Supplement: TABLE S2 [file msystems.01042-22-s0008.docx]

Table S2. qRT-PCR quantification of the gene expression on nine selected genes.

| Gene ID | Iog_2_ fold change (Δ*bcxyr1*^a^/wt^b^) | Iog_2_ fold change (Δ*bcxyr1*^a^/wt^b^) | Iog_2_ fold change (ox-*bcxyr1^c^*/wt^b^) |
| --- | --- | --- | --- |
|  | RNA-seq | qRT-PCR | qRT-PCR |
| Bcin01g02460 | -1.08 | -1.56 | 0.75 |
| Bcin01g07100 | 3.29 | 2.47 | -4.4 |
| Bcin02g07640 | 1.70 | 4.1 | 2.81 |
| Bcin02g07770 | -1.88 | -1.4 | 4.07 |
| Bcin06g05050 | -1.78 | -2.42 | 2.22 |
| Bcin07g02730 | -2.50 | -1.48 | 1.18 |
| Bcin08g02110 | -1.98 | -2.52 | 3.28 |
| Bcin14g00610 | -3.50 | -3.29 | 0.57 |
| Bcin14g05500 | -1.63 | -2.57 | -0.29 |

^a^ Gene expression level in the Δ*bcxyr1* strain.

^b^ Gene expression level in the wild type strain.

^c^ Gene expression level in the ox-*bcxyr1* strain.
