# Supplementary material for: Botrytis cinerea Transcription Factor BcXyr1 Regulates (Hemi-)Cellulase Production and Fungal Virulence
Source: mSystems. 2022 Dec 5;7(6):e01042-22. doi: 10.1128/msystems.01042-22 (PMC9765177; doi:10.1128/msystems.01042-22)
Supplement: TABLE S3 [file msystems.01042-22-s0009.docx]

Table S3. Secreted protein-encoding genes downregulated in the Δ*bcxyr1* strain.

| **Gene ID** | **Description** |
| --- | --- |
| Bcin14g00610 * | endopolygalacturonase 2, BcPg2, GH28 |
| Bcin03g01250 | predicted protein |
| Bcin07g02730 * | Pectate lyase, GH55 |
| Bcin08g02110 * | Endoglucanase B, GH5 |
| Bcin02g07770 | Necrosis inducing protein, BcNep2 |
| Bcin02g07070 | predicted protein |
| Bcin02g07470 | hypothetical protein |
| Bcin06g05050 * | Glucoamylase, CBM20 |
| Bcin01g09540 | predicted protein |
| Bcin11g02900 | Trypsin |
| Bcin14g05500 * | glucose oxidase, BcGod1 |
| Bcin08g05870 * | Alpha-fucosidase A, GH65 |
| Bcin05g03680 | predicted protein |
| Bcin15g04770 | predicted protein |
| Bcin12g02910 * | putative cellobiose dehydrogenase |
| Bcin05g03190 | hypothetical protein |
| Bcin10g01020 | hypothetical protein |
| Bcin08g05450 | predicted protein |
| Bcin06g03800 * | putative glycoside hydrolase family 16 protein |
| Bcin13g05710 * | putative galactose oxidase precursor protein, Bcgox |
| Bcin01g02460 * | protein related to plant expansins |
| Bcin01g05680 * | Probable mannosyl-oligosaccharide alpha-1,2-mannosidase 1B (Precursor), GH47 |

* CAZyme-encoding gene.
