# Supplementary material for: Botrytis cinerea Transcription Factor BcXyr1 Regulates (Hemi-)Cellulase Production and Fungal Virulence
Source: mSystems. 2022 Dec 5;7(6):e01042-22. doi: 10.1128/msystems.01042-22 (PMC9765177; doi:10.1128/msystems.01042-22)
Supplement: TABLE S4 [file msystems.01042-22-s0010.docx]

Table S4. Primers used in this study.

| Primer | Name | Purpose | Sequence (5’- 3’) |
| --- | --- | --- | --- |
| 1 | xyr1 5’for | Amplify 5’flank of *bcxyr1* | ACCACCAGAGCATCCTTGAT |
| 2 | xyr1 5’rev | Amplify 5’flank of *bcxyr1* | CATTCGCGAGGTACCGAGCTTTTGGAGTTGATGTGCAAAC |
| 3 | xyr1 3’for | Amplify 3’flank of *bcxyr1* | TCAGGAATTATTCTCACAGTCTCACAGTGCGCGATGAATT |
| 4 | xyr1 3’rev | Amplify 3’flank of *bcxyr1* | AACTCCTATTCCACAGCTGC |
| 5 | Polic-for | Amplify *hph* cassette | AGCTCGGTACCTCGCGAATG |
| 6 | ToACR rev | Amplify *hph* cassette | ACTGTGAGAATAATTCCTGAA |
| 7 | Ptrpc for | 5’ primer of Ptrpc to amplify nr cassette | AACTGATATTGAAGGAGCAT |
| 8 | xyr1 for | 5’ primer of *bcxyr1* ORF | ATGCTTTCCAATCCCCTTC |
| 9 | xyr1 ORF rev | 3’ primer of bcxyr1 ORF | CATACATCTTATCTACATACTTATAAAGCTAAACCCGTTCCAT |
| 10 | Tglu for | Amplify *Tcel5a* termination signal | TAAGTATGTAGATAAGATGTATG |
| 11 | Ptrpc-Tglu | Amplify *Tcel5a* termination signal | ATGCTCCTTCAATATCAGTTCCTCTGCAGTCGACGGG |
| 12 | xyr1-pro | Amplify 5’flank of *bcxyr1* to fuse with *bcxyr1* ORF | GAAGGGGATTGGAAAGCATTTTGGAGTTGATGTGCAAAC |
| 13 | gapdh ORF 3’ | Amplify 3’ part of *bcgapdh* ORF and its termination sequence | ATCGTAACACAGGCATATTG |
| 14 | gapdh rev | Amplify 3’ part of *bcgapdh* ORF and its termination sequence | GCACTACTACTACTCCTAAC |
| 15 | xyr1 ORF for | Amplify *bcxyr1* to fuse with H2B promoter | CCAAACAAATTTTAAATTCAAAATGCTTTCCAATCCCCTTC |
| 16 | Ph2b for | Amplify H2B promoter | TTTGAATTTAAAATTTGTTTGG |
| 17 | PtrpC-Ph2b | Amplify H2B promoter | GCTCCTTCAATATCAGTTCTAGTTTTGAAAGTTGTTTAG |
| 18 | swo 5'for | Amplify 5’flank of *Bcin01g02460* | CCAATCTCCAGAACGCAATC |
| 19 | swo 5'rev | Amplify 5’flank of *Bcin01g02460* | CATTCGCGAGGTACCGAGCTATTGATTGTAAGGGGTTGT |
| 20 | swo 3'for | Amplify 3’flank of *Bcin01g02460* | TCAGGAATTATTCTCACAGTAGTGGACATTGCTCCTCGAG |
| 21 | swo 3'rev | Amplify 3’flank of *Bcin01g02460* | TAACTTGACGCGGGTTGTCT |
| 22 | Hyg end for | Used to check homologous recombination of *hph*-containing cassette | ATTCCCAATACGAGGTCGCCAACATCTTCTTC |
| 23 | Hyg beg rev | Used to check homologous recombination of *hph*-containing cassette | GAGATGCAATAGGTCAGGCTCTCGCTGAATTC |
| 24 | NAT end for | Used to check homologous recombination of *nr*-containing cassette | TGAGCGGCCTGCAGGAATTCTAGAG |
| 25 | xyr1 5'check for | Used to check homologous recombination of *bcxyr1* locus | GTGTTTGCCATACGCCATAC |
| 26 | xyr1 3'check rev | Used to check homologous recombination of *bcxyr1* locus | ATGAGCGAATGAGCGAGCAT |
| 27 | gapdh 5'check for | Used to check homologous recombination of *bcgapdh* locus | TGTCCATGCGTGTTCCAACT |
| 28 | gapdh 3'check rev | Used to check homologous recombination of *bcgapdh* locus | AGCCAATGATTACGGAGTGC |
| 29 | swo 5'check for | Used to check homologous recombination of Bcin01g02460 locus | TCGTTTCAGAGGGATTCAG |
| 30 | swo 3'check rev | Used to check homologous recombination of Bcin01g02460 locus | TCACCAGCACCATCACTAT |
| 31 | rt-gpdh for | For Real-time PCR amplification of *bcgpdh* | CGAAGAATAGCACAAACAGCTGGAC |
| 32 | rt-gpdh rev | For Real-time PCR amplification of *bcgpdh* | CGTCACCTTATGCTTCTTGCTCC |
| 33 | rt-xyr1 for | For Real-time PCR amplification of *bcxyr1* | GATTCGGCATTGGCTTTCGTTC |
| 34 | rt-xyr1 rev | For Real-time PCR amplification of *bcxyr1* | TCAGTTTGATCATCCGGCAAGG |
| 35 | rt-01g02460F | For Real-time PCR amplification of Bcin01g02460 | GCATGCTCCTTCTCTACCTATACCC |
| 36 | rt-01g02460R | For Real-time PCR amplification of Bcin01g02460 | GGGACACTCGTCAGTAATCATAGCA |
| 37 | RT-01g07100F | For Real-time PCR amplification of Bcin01g07100 | CGTCATGTCACTCGAGTTTCTTCG |
| 38 | RT-01g07100R | For Real-time PCR amplification of Bcin01g07100 | GGACGTGATGTGCTGGAGTTGATT |
| 39 | RT-02g07640F | For Real-time PCR amplification of Bcin02g07640 | ACCACTCCATCATCTTCCGCTACT |
| 40 | RT-02g07640R | For Real-time PCR amplification of Bcin02g07640 | TCAACACCGATATTGATGGCGACG |
| 41 | RT-02g07770F | For Real-time PCR amplification of Bcin02g07770 | TGGGAAGGTGTAATTGTCTGGCTC |
| 42 | RT-02g07770R | For Real-time PCR amplification of Bcin02g07770 | GAATAGCCATCCGTGGAACAATCC |
| 43 | RT-06g05050F | For Real-time PCR amplification of Bcin06g05050 | CCTTCTCTGATTCCCGTAACCTTC |
| 44 | RT-06g05050R | For Real-time PCR amplification of Bcin06g05050 | AGTTTACAGCTGACAATGCGACGG |
| 45 | RT-07g02730F | For Real-time PCR amplification of Bcin07g02730 | AACACTCATCCAGTGGAATCTCGC |
| 46 | RT-07g02730R | For Real-time PCR amplification of Bcin07g02730 | CCTGTAGTAGGACATTGAGCAACC |
| 47 | RT-08g02110F | For Real-time PCR amplification of Bcin08g02110 | CAACACAAGAGAACGCGAACGAAC |
| 48 | RT-08g02110R | For Real-time PCR amplification of Bcin08g02110 | GGGAAACTACTCTTTGAGGGTTG |
| 49 | RT-14g00610F | For Real-time PCR amplification of Bcin14g00610 | GTGGGAAGGCCCTCTTATTTCCAT |
| 50 | RT-14g00610R | For Real-time PCR amplification of Bcin14g00610 | GGCTGAGAAGAACTTAGGCTTGAC |
| 51 | RT-14g05500F | For Real-time PCR amplification of Bcin14g05500 | CCAACCGCACCAATCTTCATCTCT |
| 52 | RT-14g05500R | For Real-time PCR amplification of Bcin14g05500 | ATATGGAGTGCTTGAGTCGTTGGG |
| 53 | Histone1F | Used to check homologous recombination of H1-GFP cassette | TCAACCGCGCCTTGAAGAAC |
| 54 | Histone1R | Used to check homologous recombination of H1-GFP cassette | GGTCTCAAAACCCTCAATTCT |
| 55 | Swo R | Used to check *bcexl1* complementation | GGGTAGAGATCCAAGTGGT |
| 56 | Swo F | Used to check bcexl1 complementation | GTCACTGCTGGAAATCTCG |
